# Supplementary material for: Decreased sound tolerance in a Canadian University Context: Associations with autistic traits, social competence, and gender in an undergraduate sample
Source: PLoS One. 2025 Nov 26;20(11):e0334689. doi: 10.1371/journal.pone.0334689 (PMC12654913; doi:10.1371/journal.pone.0334689)
Supplement: S1 Table — (PDF) [file pone.0334689.s001.pdf]

S1 Table. Pearson's correlations between MSCS subscales and each other measure used.

		MSCS Total	MSCS-SM	MSCS-SI	MSCS-EC	MSCS-SK	MSCS-VCS	MSCS-NSS	MSCS-ER
Age	*Pearson's r*	*0.015*	*-0.059*	*0.033*	*0.011*	*-0.011*	*0.022*	*0.053*	*0.035*
	p -Value	*0.496*	*0.008*	*0.137*	*0.622*	*0.609*	*0.309*	*0.016*	*0.112*
AQ	*Pearson's r*	***-0.614**	***-0.625**	***-0.498**	-0.247	-0.287	***-0.402**	***-0.458**	***-0.428**
	p -Value	**<.001**	**<.001**	**<.001**	<.001	<.001	**<.001**	**<.001**	**<.001**
MQ Total	*Pearson's r*	-0.294	-0.191	-0.207	0.012	-0.113	***-0.33**	-0.134	***-0.470**
	p -Value	<.001	<.001	<.001	0.582	<.001	**<.001**	<.001	**<.001**
MQ Severity	*Pearson's r*	-0.14	-0.061	-0.102	0.021	-0.073	-0.184	-0.077	-0.199
	p -Value	<.001	0.051	0.001	0.507	0.02	<.001	0.013	<.001
DMQ Total	*Pearson's r*	***-0.368**	-0.276	-0.283	-0.006	-0.184	***-0.361**	-0.202	***-0.476**
	p -Value	**<.001**	<.001	<.001	0.776	<.001	**<.001**	<.001	**<.001**
DMQ Coping	*Pearson's r*	-0.256	-0.189	-0.208	0.049	-0.11	-0.295	-0.143	***-0.346**
	p -Value	<.001	<.001	<.001	0.025	<.001	<.001	<.001	**<.001**
DMQ Severity	*Pearson's r*	***-0.339**	-0.26	-0.247	0.008	-0.141	***-0.348**	-0.161	***-0.496**
	p -Value	**<.001**	<.001	<.001	0.71	<.001	**<.001**	<.001	**<.001**
IHS	*Pearson's r*	***-0.387**	***-0.312**	-0.296	-0.025	-0.192	***-0.359**	-0.212	***-0.479**
	p -Value	**<.001**	**<.001**	<.001	0.257	<.001	**<.001**	<.001	**<.001**

Note: Autism Spectrum Quotient (AQ); Multidimensional Social Competence Scale (MSCS); Misophonia Questionnaire (MQ); Duke Misophonia Questionnaire (DMQ); Inventory of Hyperacusis Symptoms (IHS); MSCS Subscales: Social Motivation (SM), Social Inferencing (SI), Empathic Concern (EC), Social Knowledge (SK), Verbal Conversation Skills (VCS), Non-verbal Sending Skills (NSS), Emotional Regulation (ER). *Indicates medium (or larger) effect size (r > +/- .3)
