# Supplementary material for: Decreased sound tolerance in a Canadian University Context: Associations with autistic traits, social competence, and gender in an undergraduate sample
Source: PLoS One. 2025 Nov 26;20(11):e0334689. doi: 10.1371/journal.pone.0334689 (PMC12654913; doi:10.1371/journal.pone.0334689)
Supplement: S2 Table — Note * indicating Z scores ±1.96 that demonstrate statistically significant differences. Note: Reduced sample size due to survey error. (PDF) [file pone.0334689.s002.pdf]

S2 Table. Chi-square test of association for gender and Misophonia Questionnaire diagnosis. Note * indicating Z scores ±1.96 that demonstrate statistically significant differences. Note: Reduced sample size due to survey error.

		Female	Male	Non-Cisgendered
Non-Clinical				
	Count	671*	184*	18*
	Percent	86.0%	96.8%	64.3%
	Expected Count	682	166	24
	Adjusted Residual	-2.6	4.3	-3.8
Clinical				
	Count	109*	6*	10*
	Percent	14.0%	3.2%	35.7%
	Expected Count	98	24	4
	Adjusted Residual	2.6	-4.3	3.8
