# Supplementary material for: Decreased sound tolerance in a Canadian University Context: Associations with autistic traits, social competence, and gender in an undergraduate sample
Source: PLoS One. 2025 Nov 26;20(11):e0334689. doi: 10.1371/journal.pone.0334689 (PMC12654913; doi:10.1371/journal.pone.0334689)
Supplement: S3 Table — Note * indicating Z scores ±1.96 that demonstrate statistically significant differences. (PDF) [file pone.0334689.s003.pdf]

S3 Table. Chi-square test of association for gender and Duke Misophonia Questionnaire diagnosis. Note * indicating Z scores ±1.96 that demonstrate statistically significant differences.

		Female	Male	Non-Cisgendered
Non-Clinical				
	Count	1276*	411*	19*
	Percent	79.8%	94.5%	42.2%
	Expected Count	1312	357	37
	Adjusted Residual	-4.9	7.6	-7.0
Clinical				
	Count	324*	24*	26*
	Percent	20.3%	5.5%	57.8%
	Expected Count	288	78	8
	Adjusted Residual	4.9	-7.6	7.0
