# Supplementary material for: Decreased sound tolerance in a Canadian University Context: Associations with autistic traits, social competence, and gender in an undergraduate sample
Source: PLoS One. 2025 Nov 26;20(11):e0334689. doi: 10.1371/journal.pone.0334689 (PMC12654913; doi:10.1371/journal.pone.0334689)
Supplement: S4 Table — Note * indicating Z scores ±1.96 that demonstrate statistically significant differences. (PDF) [file pone.0334689.s004.pdf]

S4 Table. **Chi-square test of association for gender and Duke-Vanderbilt Misophonia Screening Questionnaire diagnosis.** Note * indicating Z scores ±1.96 that demonstrate statistically significant differences.

		Female	Male	Non-Cisgendered
Non-Clinical				
	Count	1121*	390*	14*
	Percent	70.0%	89.7%	31.1%
	Expected Count	1173	318	33
	Adjusted Residual	-6.1	8.7	-6.5
Sub-clinical				
	Count	251*	27*	7*
	Percent	15.7%	6.2%	15.6%
	Expected Count	219	60	6
	Adjusted Residual	4.8	-5.1	0.4
Clinical				
	Count	228*	18*	24*
	Percent	14.3%	4.1%	53%
	Expected Count	208	56	6
	Adjusted Residual	3.1	-6.2	8.1
