# Supplementary material for: Decreased sound tolerance in a Canadian University Context: Associations with autistic traits, social competence, and gender in an undergraduate sample
Source: PLoS One. 2025 Nov 26;20(11):e0334689. doi: 10.1371/journal.pone.0334689 (PMC12654913; doi:10.1371/journal.pone.0334689)
Supplement: S5 Table — Note * indicating Z scores ±1.96 that demonstrate statistically significant differences. (PDF) [file pone.0334689.s005.pdf]

S5 Table. Chi-square test of association for gender and Inventory of Hyperacusis Symptoms diagnosis. Note * indicating Z scores ±1.96 that demonstrate statistically significant differences.

		Female	Male	Non-Cisgendered
Non-Clinical				
	Count	1307*	405*	24*
	Percent	81.7%	93.1%	53.3%
	Expected Count	1335	363	37
	Adjusted Residual	-4.0	6.1	-5.5
Clinical				
	Count	293*	30*	21*
	Percent	18.3%	6.9%	46.7%
	Expected Count	265	72	7
	Adjusted Residual	4.0	-6.1	5.5
