# Supplementary material for: Decreased sound tolerance in a Canadian University Context: Associations with autistic traits, social competence, and gender in an undergraduate sample
Source: PLoS One. 2025 Nov 26;20(11):e0334689. doi: 10.1371/journal.pone.0334689 (PMC12654913; doi:10.1371/journal.pone.0334689)
Supplement: S6 Table — Note * indicating Z scores ±1.96 that demonstrate statistically significant differences. (PDF) [file pone.0334689.s006.pdf]

S6 Table. Chi-square test of association for gender and Autism Quotient diagnosis. Note * indicating Z scores ±1.96 that demonstrate statistically significant differences.

		Female	Male	Non-Cisgendered
Non-Clinical				
	Count	1419*	405*	25*
	Percent	88.7%	93.1%	56.0%
	Expected Count	1422	387	40
	Adjusted Residual	-0.5	3.1	-7.2
Clinical				
	Count	181*	30*	20*
	Percent	11.3%	6.9%	44.4%
	Expected Count	178	48	5
	Adjusted Residual	0.5	-3.1	7.2
