# Supplementary material for: Decreased sound tolerance in a Canadian University Context: Associations with autistic traits, social competence, and gender in an undergraduate sample
Source: PLoS One. 2025 Nov 26;20(11):e0334689. doi: 10.1371/journal.pone.0334689 (PMC12654913; doi:10.1371/journal.pone.0334689)
Supplement: S1 Appendix — (PDF) [file pone.0334689.s007.pdf]

Psychometric Properties of Study Measures

Autism Spectrum Quotient (AQ)

Internal consistency of the AQ total score has been determined to range from fair (Cronbach's $\alpha = 0.74$)[1] to good (Cronbach's $\alpha = 0.82$)[2].

Multidimensional Social Competence Scale (MSCS)

The MSCS was first designed and validated by Yager and Iarocci [3] as a parent-rating scale in a sample of adolescents. It was then validated for self-report in a sample of 1178 young adults by Trevisan and colleagues [4]. Good internal consistency on this self-report version of the MSCS was found for each subscale using Cronbach's alpha: the Emotion Regulation ($\alpha = 0.83$), Verbal Conversation Skills ($\alpha = 0.79$), Social Knowledge ($\alpha = 0.74$), Social Inferencing ($\alpha = 0.79$), Social Motivation ($\alpha = 0.86$), Nonverbal Sending Skills ($\alpha = 0.80$), and Empathic Concern ($\alpha = 0.84$) subscales, as well as the Full Scale ($\alpha = 0.80$). All subscales and the total scale were additionally found to have good convergent and discriminant validity.

Misophonia Questionnaire (MQ)

The MQ was designed by Wu and colleagues [5] and validated on a sample of 483 university students. In their study, the MQ total score demonstrated good discriminant validity with the Adult Sensory Questionnaire (ASQ)[6] which assesses the general presence of sound sensitivities. In their sample, Cronbach's alphas showed good internal consistency for the Misophonia Symptom Scale (0.86), Misophonia Emotions and Behaviors Scale (0.86) and for the total MQ score (0.89).

Duke Misophonia Questionnaire (DMQ)

The DMQ was developed and validated by Rosenthal and colleagues [7] on a sample of 424 adults. Strong correlations were found between each of the Symptoms and Coping composite scales and the Beliefs scale (Pearson's r's >0.696), demonstrating good convergent validity. Correlations between DMQ scales and existing misophonia measures such as the MQ [5] were also found to be strong, also demonstrating good convergent validity. DMQ Symptoms scale scores demonstrated a good ability to discriminate clinical and subclinical cases of misophonia when compared with the MQ severity scale.

Duke-Vanderbilt Misophonia Screening Questionnaire (DVMSQ)

The DVMSQ was developed and validated by Williams and colleagues [8] with one sample of 1403 general population adults and one sample of 936 autistic adults. Pearson's correlations between the DVMSQ total score and other validated measures such as the DMQ [7] Symptoms scale were high ($r = 0.80$), suggesting generally good convergent validity.

Inventory of Hyperacusis Symptoms (HIS)

The IHS was created and validated by Greenberg and Carlos [9] with a sample of 324 non-autistic adults with varying levels of auditory sensitivities. This measure had a high internal consistency with a Cronbach's alpha of 0.93.

References

[1] Gardiner E, Iarocci G. Students with autism spectrum disorder in the university context: Peer acceptance predicts intention to volunteer. Journal of autism and developmental disorders. 2014 May;44:1008-17.

[2] Hurst RM, Mitchell JT, Kimbrel NA, Kwapil TK, Nelson-Gray RO. Examination of the reliability and factor structure of the Autism Spectrum Quotient (AQ) in a non-clinical sample. Personality and Individual Differences. 2007 Nov 1;43(7):1938-49.

[3] Yager J, Iarocci G. The development of the multidimensional social competence scale: A standardized measure of social competence in autism spectrum disorders. Autism Research. 2013 Dec;6(6):631-41.

[4] Trevisan DA, Tafreshi D, Slaney KL, Yager J, Iarocci G. A psychometric evaluation of the Multidimensional Social Competence Scale (MSCS) for young adults. PloS one. 2018 Nov 2;13(11):e0206800.

[5] Wu MS, Lewin AB, Murphy TK, Storch EA. Misophonia: incidence, phenomenology, and clinical correlates in an undergraduate student sample. Journal of clinical psychology. 2014 Oct;70(10):994-1007.

[6] Kinnealey, M., & Oliver, B. (2002). Adult Sensory Questionnaire. Philadelphia, PA: Temple University, College of Allied Health Professionals

[7] Rosenthal MZ, Anand D, Cassiello-Robbins C, Williams ZJ, Guetta RE, Trumbull J, Kelley LD. Development and initial validation of the duke misophonia questionnaire. Frontiers in psychology. 2021 Sep 29;12:709928.

[8] Williams ZJ, Cascio CJ, Woynaroski TG. Psychometric validation of a brief self-report measure of misophonia symptoms and functional impairment: The duke-vanderbilt misophonia screening questionnaire. Frontiers in Psychology. 2022 Jul 22;13:897901.

[9] Greenberg B, Carlos M. Psychometric properties and factor structure of a new scale to measure hyperacusis: introducing the inventory of hyperacusis symptoms. Ear and hearing. 2018 Sep 1;39(5):1025-34.
