# Supplementary material for: Decreased sound tolerance in a Canadian University Context: Associations with autistic traits, social competence, and gender in an undergraduate sample
Source: PLoS One. 2025 Nov 26;20(11):e0334689. doi: 10.1371/journal.pone.0334689 (PMC12654913; doi:10.1371/journal.pone.0334689)
Supplement: S1 Data — (PDF) [file pone.0334689.s008.pdf]

Participant	Year of Bir	IHS Total	DMQ-Sev	DMQ-Cop	AQ Total	Gender
1	1996	33	5	17	14	Male
2	2003	43	32	41	33	Female
3	2002	36	12	17	23	Female
4	2003	78	67	54	36	Female
5	2001	32	13	16	19	Female
6	2003	34	5	15	19	Female
7	2005	29	9	9	15	Female
8	2005	34	20	29	24	Female
9	2004	54	44	32	28	Female
10	2005	24	4	1	18	Male
11	2002	27	1	9	12	Female
12	2004	26	0	0	11	Female
13	2005	63	47	39	32	Female
14	2004	28	10	34	8	Female
15	2004	27	6	6	14	Female
16	2005	25	0	0	17	Female
17	2004	29	2	1	14	Male
18	2005	86	75	75	29	Female
19	2005	60	23	10	24	Female
20	2005	34	17	20	14	Female
21	2004	60	49	34	21	Female
22	2003	55	63	47	20	Female
23	2005	28	18	8	8	Male
24	2004	25	2	0	15	Female
25	2005	26	0	3	18	Female
26	2002	60	37	50	21	Female
27	2004	61	57	61	27	Female
28	2004	51	24	28	26	Male
29	2004	25	0	0	8	Male
30	2004	30	23	4	19	Female
31	2005	40	25	17	18	Female
32	2005	53	29	47	13	Male
33	2004	29	2	6	21	Female
34	2004	57	44	44	13	Female
35	2005	43	12	23	21	Female
36	2004	25	0	0	8	Female
37	2003	49	32	46	17	Female
38	2001	45	32	21	24	Female
39	2002	31	20	20	12	Female
40	2003	26	0	2	20	Male
41	2004	58	29	23	24	Female

42	2002	47	24	42	28	Female
43	2005	41	50	45	29	Female
44	2004	25	3	0	12	Female
45	2003	69	70	47	20	Female
46	2005	29	3	5	14	Female
47	2005	31	9	11	15	Female
48	2004	33	9	10	12	Female
49	2000	84	45	45	31	Other (please ty
50	2005	32	23	12	17	Female
51	2003	73	51	46	24	Female
52	2005	58	33	28	25	Female
53	2005	31	4	11	15	Female
54	2003	26	1	13	18	Female
55	2004	25	2	1	9	Male
56	2003	38	31	41	22	Female
57	2003	26	1	4	13	Female
58	2003	30	3	3	23	Female
59	2003	27	0	2	14	Male
60	2003	25	1	0	16	Female
61	2023	93	74	52	36	Other (please ty
62	2001	70	43	41	23	Female
63	1999	48	15	45	18	Female
64	2005	36	7	14	14	Male
65	2005	38	44	38	24	Female
66	2005	66	44	51	17	Female
67	2004	46	21	50	19	Female
68	2004	32	10	32	13	Male
69	2004	29	30	21	25	Female
70	2001	62	42	35	18	Female
71	1999	67	46	47	21	Female
72	2005	36	27	57	15	Female
73	2003	26	4	15	16	Male
74	2005	35	7	9	21	Female
75	2005	30	44	27	13	Female
76	2005	44	29	35	20	Female
77	2005	36	6	14	17	Female
78	2004	39	28	28	22	Female
79	2005	49	46	26	19	Female
80	2002	25	0	8	26	Male
81	2002	27	8	0	24	Female
82	2005	55	66	52	22	Female
83	2000	27	4	10	6	Female

84	2005	55	31	13	36	Female
85	2005	31	6	10	22	Male
86	2004	26	3	0	15	Female
87	2000	53	23	24	22	Female
88	2005	66	50	34	38	Female
89	2004	31	30	20	19	Female
90	2003	51	59	24	29	Other (please ty
91	2005	25	58	13	20	Male
92	2004	41	42	61	24	Female
93	2005	35	40	46	25	Female
94	2005	36	37	21	21	Female
95	2001	45	26	40	16	Female
96	2005	53	57	54	28	Female
97	1981	33	9	13	13	Female
98	2004	55	46	52	23	Female
99	2002	25	11	17	23	Female
100	2005	64	55	56	18	Female
101	2005	78	50	55	29	Female
102	2004	64	44	30	26	Female
103	2003	32	16	12	19	Female
104	2005	71	69	59	33	Female
105	2005	41	15	25	27	Female
106	2005	34	21	33	13	Female
107	2001	66	48	31	22	Female
108	1992	52	31	42	21	Female
109	2001	46	26	21	28	Female
110	2001	37	26	4	19	Female
111	2004	33	12	6	18	Female
112	2003	51	38	29	18	Female
113	2005	43	15	23	34	Female
114	2003	50	41	42	26	Female
115	2004	27	3	3	16	Female
116	1964	31	2	4	33	Male
117	2004	50	55	13	20	Female
118	2003	29	15	15	17	Female
119	2000	66	44	40	25	Female
120	2004	25	0	3	14	Male
121	2005	52	25	21	22	Male
122	1991	25	5	10	16	Male
123	2005	28	5	4	24	Female
124	2003	39	15	2	35	Female
125	2003	27	4	6	11	Female

126	2003	81	58	60	32	Female
127	2003	45	49	30	18	Female
128	2001	25	14	0	20	Female
129	2003	64	49	46	28	Female
130	2005	25	0	0	10	Female
131	2005	53	3	38	19	Female
132	2003	67	39	39	31	Female
133	2004	47	48	31	18	Female
134	2003	45	27	20	16	Female
135	2004	27	4	15	15	Male
136	2005	54	56	32	24	Female
137	2005	26	0	1	12	Female
138	1985	41	20	25	17	Female
139	2003	36	23	9	27	Female
140	2005	52	52	31	17	Female
141	2000	35	0	0	20	Male
142	2004	50	57	36	18	Female
143	2005	35	5	0	17	Female
144	2023	41	39	18	24	Female
145	2005	68	46	43	22	Female
146	2004	39	11	7	20	Male
147	2005	56	66	25	20	Female
148	2004	50	18	44	22	Male
149	2005	55	63	48	16	Female
150	2003	27	6	0	22	Male
151	2002	38	16	10	20	Female
152	2005	74	51	53	30	Female
153	2005	28	19	33	23	Female
154	2002	29	2	8	21	Male
155	2004	25	0	3	22	Male
156		47	22	16	19	Female
157	2003	35	25	30	26	Female
158	2005	43	18	29	17	Female
159	2004	52	44	43	33	Female
160	2003	30	9	6	19	Female
161	2004	41	19	26	18	Female
162	2002	42	36	30	27	Male
163	1984	25	2	2	4	Male
164	2003	61	38	33	19	Female
165	2005	30	32	32	23	Male
166	2004	31	8	10	13	Female
167	2003	25	0	1	14	Female

168	2002	28	2	2	25	Male
169	2004	58	15	18	29	Female
170	2003	31	1	25	15	Female
171	2003	42	46	30	27	Female
172	2002	59	40	49	23	Female
173	2004	58	36	26	31	Female
174	2005	37	25	7	19	Female
175	2002	92	78	44	34	Female
176	2004	73	43	47	26	Female
177	2004	30	12	3	9	Female
178	2003	25	6	0	11	Female
179	2002	81	58	50	22	Female
180	2004	49	34	27	19	Female
181	2002	21	3	4	21	Male
182	2005	67	52	54	18	Female
183	2003	50	36	57	14	Female
184	2004	45	34	30	17	Female
185	2005	25	11	17	23	Female
186	2004	43	15	20	24	Female
187	2004	37	47	23	19	Female
188	2005	29	8	1	14	Male
189	1966	67	34	8	29	Female
190	2000	31	49	13	14	Female
191	2004	64	57	28	16	Female
192	2005	60	42	31	19	Female
193	2023	43	21	33	22	Male
194	2004	25	1	0	23	Male
195	2005	35	6	3	26	Female
196	2002	40	32	25	10	Female
197	2002	39	7	4	26	Female
198	2005	38	19	16	26	Male
199	2004	50	22	40	16	Male
200	2004	60	45	50	24	Male
201	2005	43	24	19	25	Female
202	2005	79	63	32	37	Female
203	2001	62	47	37	29	Female
204	2005	52	25	19	14	Female
205	2005	25	8	5	14	Female
206	2002	31	9	9	19	Female
207	1995	58	54	45	24	Transgender
208	2004	57	44	62	23	Female
209	2005	30	14	30	16	Female

210	2005	40	47	29	23	Other (please ty
211	2002	25	0	0	15	Female
212	2005	34	10	9	19	Female
213	2003	31	22	23	20	Female
214	2005	37	16	22	14	Female
215	2003	27	17	8	15	Male
216	2005	25	2	0	9	Female
217	2005	30	16	16	26	Female
218	2005	55	20	34	26	Female
219	2005	25	2	1	10	Female
220	2003	40	21	30	17	Male
221	2004	35	6	8	20	Female
222	2005	26	4	2	23	Female
223	2001	47	30	32	12	Male
224	2005	41	36	27	25	Female
225	2005	32	3	5	24	Male
226	2005	47	38	39	18	Female
227	2005	27	4	3	15	Female
228	2003	31	29	23	19	Female
229	1992	38	9	6	16	Female
230	2005	59	44	22	20	Female
231	2005	28	64	40	29	Female
232	2004	25	0	0	19	Male
233	2004	40	14	2	28	Female
234	2002	25	36	49	21	Female
235	2023	39	15	16	20	Male
236	2003	28	8	5	9	Female
237	2005	36	19	13	21	Female
238	2005	25	1	14	8	Female
239	2005	25	17	0	33	Female
240	2003	42	55	34	14	Female
241	2003	46	14	33	13	Female
242	2005	81	66	47	17	Female
243	2002	90	62	60	38	Transgender
244	2005	26	10	0	14	Female
245	2003	66	43	26	20	Female
246	2004	26	8	1	20	Female
247	2005	50	17	23	14	Male
248	2001	36	18	17	31	Female
249	2004	30	12	3	12	Female
250	2005	24	3	8	18	Male
251	2004	40	25	35	28	Female

252	2003	27	0	1	13	Female
253	2005	32	9	20	15	Female
254	2002	26	2	3	11	Female
255	2005	55	28	31	18	Female
256	2005	41	39	33	23	Female
257	2005	31	21	7	21	Female
258	2005	72	61	40	23	Female
259	2004	45	27	21	22	Female
260	2004	65	42	37	27	Female
261	2004	26	3	1	11	Male
262	2004	66	32	44	21	Female
263	2003	39	14	28	19	Male
264	2003	40	33	13	27	Female
265	2005	47	25	13	28	Female
266	1999	37	11	10	30	Female
267	2004	32	24	29	12	Female
268	2004	70	55	40	35	Female
269	2004	46	39	40	24	Female
270	2002	33	35	36	16	Female
271	2005	25	3	3	12	Female
272	2003	27	2	3	6	Female
273	2003	25	8	1	16	Female
274	2004	71	66	24	16	Female
275	2005	31	1	5	18	Female
276	2004	61	32	35	30	Female
277	2004	35	36	21	27	Female
278	2004	30	11	8	20	Female
279	2005	53	52	34	18	Female
280	2004	90	81	60	25	Female
281	2003	66	58	37	18	Female
282	2004	25	6	7	17	Female
283	2003	30	2	27	30	Male
284	2004	35	32	19	20	Female
285	2003	38	26	10	17	Male
286	2004	26	11	6	14	Female
287	2005	29	14	17	14	Female
288	2002	25	12	3	16	Female
289	2001	90	71	46	45	Female
290	2004	33	8	21	27	Female
291	2003	67	56	51	26	Female
292	2004	30	26	8	15	Female
293	2004	35	10	15	15	Female

294	2005	32	15	14	17	Female
295	1999	64	29	43	21	Female
296	2005	33	38	18	14	Female
297	2003	27	8	3	14	Female
298	2002	25	0	0	7	Male
299	2005	46	18	11	21	Female
300	2002	62	42	43	20	Female
301	2003	25	0	0	21	Female
302	2004	25	1	0	22	Female
303	2005	26	3	4	25	Female
304	2003	29	21	10	15	Female
305	2003	33	27	20	17	Female
306	2003	50	39	48	20	Female
307	2004	37	50	55	22	Male
308	2003	25	0	0	19	Female
309	2003	34	21	24	24	Female
310	2003	58	10	24	14	Female
311	2004	31	18	6	12	Female
312	2005	25	0	0	16	Female
313	2005	77	59	62	19	Female
314	2001	61	30	39	27	Female
315	2005	32	10	0	18	Female
316	2005	38	27	21	18	Female
317	2005	54	48	47	29	Female
318	2005	79	72	48	40	Female
319	2004	45	40	28	19	Female
320	2004	100	92	67	50	Female
321	2004	44	15	9	16	Female
322	2005	33	3	9	17	Female
323	2005	25	0	0	10	Female
324	2005	39	37	21	27	Female
325	2004	36	22	34	25	Female
326	2004	53	25	47	26	Female
327	2001	25	3	2	19	Male
328	2004	24	12	16	23	Male
329	2003	44	24	30	23	Female
330	2005	29	17	26	18	Female
331	2003	33	18	17	27	Male
332	2005	50	48	48	18	Female
333	2005	33	15	10	17	Female
334	2002	37	58	32	20	Female
335	2003	47	38	28	17	Female

336	2005	72	56	36	24	Female
337	2003	31	8	9	15	Female
338	2004	28	7	4	22	Male
339	2005	44	39	51	41	Female
340	2005	41	26	12	16	Female
341	2002	42	24	26	29	Female
342	2004	47	31	32	18	Female
343	2005	27	3	10	23	Female
344	2004	43	17	32	17	Female
345	2003	53	37	50	14	Female
346	2005	67	58	48	26	Female
347	2003	28	7	7	13	Male
348	2001	46	41	22	30	Female
349	2004	62	40	31	21	Female
350	2003	30	8	2	12	Female
351	2000	53	28	20	33	Male
352	2004	33	21	16	20	Male
353	2005	31	11	12	20	Male
354	2003	31	39	39	13	Female
355	2005	52	57	59	25	Female
356	2001	85	76	49	33	Female
357	2005	33	12	14	21	Female
358	2005	27	2	0	12	Female
359	2002	29	1	5	7	Female
360	2005	50	20	8	16	Female
361	2003	36	16	22	24	Male
362	2004	36	17	35	18	Female
363	2002	25	9	8	14	Female
364	2003	30	0	4	23	Female
365	2003	94	55	17	34	Female
366	2004	45	24	17	15	Female
367	2005	30	29	4	14	Female
368	2004	70	60	53	24	Female
369	2005	29	2	2	20	Female
370	2005	31	18	20	16	Female
371	2004	29	25	24	22	Female
372	2005	38	12	10	17	Female
373	2004	33	14	2	26	Female
374	2003	27	0	8	22	Male
375	2003	44	31	17	18	Female
376	2003	29	14	24	15	Female
377	2003	28	9	21	16	Male

378	2023	45	26	29	14	Female
379	2005	56	63	48	19	Female
380	2000	91	43	82	26	Female
381	2004	32	25	22	28	Female
382	2005	58	52	52	23	Female
383	2004	40	39	26	31	Female
384	2004	48	43	43	17	Female
385	2005	37	40	26	10	Female
386	2004	34	26	18	13	Female
387	2004	43	29	22	26	Male
388	2004	49	39	46	35	Female
389	2005	38	38	39	28	Female
390	1999	38	19	52	17	Male
391	2004	31	9	20	8	Female
392	2005	43	11	12	20	Female
393	2001	41	40	34	28	Female
394	2004	47	35	34	23	Female
395	2004	38	35	21	18	Female
396	2003	30	2	15	13	Female
397	2003	72	46	46	26	Female
398	2002	54	31	42	22	Transgender
399	2005	29	3	2	16	Male
400	2004	54	56	39	21	Transgender
401	2023	58	26	34	21	Female
402	2004	58	36	29	41	Female
403	2005	25	1	0	10	Female
404	2005	31	0	0	17	Female
405	2005	35	49	51	18	Female
406	2005	25	0	0	11	Male
407	2001	26	6	0	21	Female
408	2005	40	11	35	10	Female
409	1994	24	10	12	16	Female
410	2004	32	8	11	17	Female
411	2002	25	0	0	15	Female
412	2004	29	13	4	14	Female
413	2004	34	24	26	21	Male
414	2003	29	3	19	22	Male
415	2003	29	6	1	12	Male
416	2001	57	34	41	21	Female
417	2005	29	11	12	12	Female
418	2004	62	46	42	22	Female
419	2005	46	47	34	15	Other (please ty

420	2005	27	4	8	14	Male
421	2004	28	6	17	17	Male
422	2003	27	24	34	17	Male
423	2003	39	48	39	21	Female
424	2002	52	42	34	25	Female
425	2005	53	31	34	13	Female
426	2005	37	32	44	16	Female
427	2003	25	1	13	24	Female
428	2005	33	40	23	33	Female
429	2002	48	28	35	19	Female
430	2002	45	19	22	14	Female
431	2005	25	0	0	16	Female
432	2004	30	0	0	24	Female
433	2003	50	11	28	29	Female
434	2005	44	23	12	15	Female
435	2001	54	32	57	24	Female
436	2005	41	53	17	19	Female
437	2003	32	9	8	16	Female
438	2005	25	8	0	14	Female
439	2005	32	6	3	15	Female
440	2005	45	39	22	16	Female
441	2002	38	31	32	18	Female
442	2001	34	27	12	19	Female
443	2005	38	23	28	15	Female
444	2003	46	37	31	21	Female
445	2002	37	34	28	22	Female
446	2003	26	4	11	19	Female
447	2005	66	64	54	18	Female
448	2004	32	38	22	20	Male
449	2004	63	70	30	25	Female
450	2004	36	33	5	15	Female
451	2004	57	52	64	22	Female
452	2004	25	0	0	5	Female
453	2003	34	32	39	26	Female
454	2002	56	21	23	23	Female
455	2004	33	16	14	21	Female
456	2005	25	0	10	13	Male
457	2004	38	47	16	15	Female
458	2004	25	50	0	31	Female
459	2005	42	5	10	24	Female
460	2005	25	0	0	17	Female
461	2004	29	9	0	31	Female

462	2004	30	5	7	11	Female
463	2004	42	16	22	14	Female
464	2002	47	42	56	20	Female
465	2003	61	31	43	27	Female
466	2004	27	2	6	16	Female
467	2005	64	46	78	21	Female
468	2003	31	13	18	19	Male
469	2004	49	15	17	26	Female
470	2005	38	58	26	14	Female
471	2004	30	8	12	15	Female
472	2005	54	57	48	17	Female
473	2004	30	1	6	21	Female
474	2005	34	9	12	18	Male
475	2005	25	0	0	8	Male
476	2005	36	17	23	22	Female
477	2006	40	31	51	28	Male
478	2005	59	74	42	31	Female
479	2005	34	18	19	9	Male
480	2004	25	2	0	9	Male
481	2005	28	11	3	18	Female
482	2005	35	14	11	17	Female
483	2002	54	42	36	16	Female
484	2002	25	2	4	15	Male
485	2003	27	8	12	20	Male
486	2004	80	73	43	27	Female
487	2004	24	16	29	16	Male
488	2004	30	6	15	23	Female
489	2004	36	21	23	27	Female
490	2004	60	48	52	23	Female
491	2005	25	7	0	7	Male
492	2005	49	27	20	33	Prefer not to ans
493	2001	34	4	11	19	Female
494	2004	35	25	38	16	Female
495	2004	60	37	47	21	Female
496	2005	31	10	10	16	Male
497	2005	49	23	32	19	Male
498	2001	31	9	10	16	Female
499	2003	31	13	22	23	Female
500	2002	44	12	12	16	Female
501	2003	51	9	35	23	Male
502	2005	34	20	22	15	Female
503	2005	34	22	31	17	Female

504	2005	43	17	25	15	Female
505	2004	27	7	0	19	Female
506	2002	41	42	11	28	Female
507	2005	29	15	29	16	Female
508	2003	33	16	16	24	Male
509	2002	75	60	42	29	Female
510	2002	62	49	36	25	Male
511	2004	25	16	13	16	Male
512	2002	51	47	25	23	Male
513	2005	73	36	43	38	Female
514	2005	53	42	34	24	Female
515	2004	43	25	23	23	Female
516	2005	25	0	0	14	Female
517	2005	38	54	28	11	Female
518	2000	58	46	44	24	Other (please ty
519	2003	48	20	42	19	Female
520	2005	32	6	6	20	Female
521	2003	25	0	4	11	Female
522	2004	56	60	7	19	Female
523	2005	37	16	31	33	Female
524	2004	64	31	35	31	Female
525	2002	35	40	29	6	Female
526	2004	26	6	5	5	Female
527	2002	26	4	4	9	Female
528	2004	52	28	43	27	Female
529	2004	60	49	39	23	Female
530	2004	29	0	16	21	Female
531	2003	28	5	0	15	Female
532	2003	31	20	14	17	Male
533	2003	38	41	41	7	Female
534	2002	39	23	15	19	Female
535	2002	56	43	40	26	Female
536	2004	45	19	35	17	Female
537	2003	43	18	23	21	Female
538	2004	29	11	4	13	Male
539	2004	26	5	1	17	Female
540	2002	28	13	27	18	Female
541	2004	49	40	36	22	Male
542	2003	64	52	51	22	Female
543	2005	54	32	26	19	Female
544	2005	39	50	6	22	Female
545	2005	37	26	9	19	Female

546	2004	28	1	4	27	Female
547	2005	47	26	25	20	Female
548	2004	29	6	5	24	Male
549	2005	30	28	28	19	Female
550	2004	68	63	46	32	Female
551	2005	25	0	0	18	Female
552	2002	57	51	59	31	Female
553	2004	25	0	0	20	Female
554	2005	24	19	7	19	Female
555	2004	69	48	31	34	Female
556	2004	25	0	0	4	Female
557	2001	84	76	46	38	Female
558	2004	64	44	33	31	Female
559	2005	33	16	12	18	Male
560	2001	51	31	19	39	Female
561	2004	32	6	3	21	Female
562	2002	53	7	21	42	Female
563	2004	24	0	0	18	Male
564	2005	25	3	3	15	Female
565	1999	28	0	0	16	Female
566	2003	71	34	42	27	Female
567	2004	35	14	5	16	Female
568	2004	51	36	27	14	Female
569	1995	61	28	35	18	Female
570	2005	33	11	11	15	Female
571	2005	25	2	2	15	Female
572	1998	32	25	30	16	Female
573	2004	25	0	0	15	Female
574	2002	46	32	19	21	Female
575	1995	74	43	25	24	Female
576	2005	54	41	49	27	Other (please ty
577	2005	27	3	5	11	Female
578	2004	32	7	16	14	Female
579	2005	27	1	1	14	Female
580	2003	28	24	12	5	Female
581	2003	25	8	12	15	Female
582	2004	32	25	23	12	Female
583	2004	31	8	10	23	Male
584	2005	60	52	47	21	Female
585	2005	42	28	22	17	Female
586	2003	35	49	55	15	Female
587	2005	27	0	7	5	Female

588	2005	27	2	6	15	Female
589	2005	35	9	14	16	Female
590	2004	70	65	21	25	Female
591	2005	26	0	0	22	Male
592	2002	44	32	31	27	Female
593	2003	33	23	23	30	Female
594	2005	33	24	28	15	Female
595	2023	35	32	40	14	Female
596	2000	42	22	29	19	Female
597	2004	26	4	34	15	Female
598	2004	26	1	6	13	Female
599	2003	48	27	24	23	Female
600	2004	62	64	56	21	Female
601	2005	25	0	0	15	Female
602	2004	33	6	6	21	Female
603	2004	43	27	31	19	Male
604	2005	49	38	50	20	Female
605	2005	28	15	18	19	Female
606	2001	40	24	20	21	Other (please ty
607	2004	27	4	10	15	Female
608	2004	78	54	64	32	Female
609	2002	26	1	2	19	Female
610	2005	44	43	32	24	Female
611	2005	68	34	63	20	Male
612	2003	27	5	6	10	Male
613	2004	37	14	13	19	Female
614	2005	31	29	77	12	Female
615	2005	25	7	2	25	Female
616	2004	26	1	4	20	Female
617	2005	29	8	4	23	Female
618	2002	27	1	1	25	Female
619	2001	34	17	31	17	Other (please ty
620	2005	45	37	28	20	Female
621	2005	39	49	51	17	Female
622	2005	58	50	52	15	Female
623	2002	33	34	9	14	Female
624	2005	36	14	25	30	Transgender
625	2005	48	38	18	26	Female
626	2005	49	37	33	16	Female
627	2005	44	15	13	29	Female
628	2004	35	25	19	13	Male
629	2005	34	12	49	13	Female

630	2005	30	11	8	18	Female
631	2004	29	5	0	11	Male
632	2005	45	14	33	35	Male
633	2004	54	32	32	23	Male
634	2002	34	31	4	21	Female
635	2001	35	26	18	19	Female
636	2001	25	8	9	20	Female
637	2003	67	32	40	26	Female
638	2005	31	14	4	27	Female
639	2005	32	31	34	26	Male
640	2004	45	42	39	28	Female
641	2004	34	20	5	28	Female
642		44	25	24	13	Female
643	2005	53	64	63	19	Female
644	2004	26	11	15	13	Male
645	2005	32	18	21	14	Female
646	2003	38	18	11	18	Male
647	2003	25	0	0	12	Male
648	2005	30	6	4	12	Female
649	2005	30	7	10	10	Female
650	2004	59	41	45	24	Female
651	2005	38	5	25	16	Male
652	2005	39	46	43	21	Female
653	2005	42	19	11	30	Female
654	2004	33	7	14	28	Female
655	2004	43	33	7	16	Female
656	2005	34	21	23	29	Male
657	2005	47	40	33	34	Female
658	2005	35	13	33	22	Male
659	2005	26	3	4	12	Female
660	2005	23	5	7	15	Female
661	2004	34	23	24	18	Female
662	2004	30	31	40	15	Female
663	2005	38	33	48	19	Female
664	2005	29	13	14	21	Female
665	2003	46	28	25	21	Male
666	2004	25	1	2	15	Female
667	2004	28	2	1	13	Female
668	2004	45	41	9	10	Male
669	2005	39	23	31	25	Female
670	2005	49	42	38	22	Female
671	2005	65	23	38	26	Female

672	2005	26	1	0	26	Female
673	2002	66	65	45	30	Female
674	2005	54	41	29	23	Female
675	2002	36	20	12	18	Female
676	2004	29	2	7	19	Male
677	2004	64	36	10	17	Female
678	2004	43	41	26	18	Female
679	2002	28	28	56	18	Female
680	2005	39	37	24	17	Female
681	2005	25	0	0	7	Male
682	2002	42	37	44	19	Female
683	1999	30	7	5	28	Male
684	2004	29	10	8	18	Female
685	2005	40	13	13	14	Male
686	2005	54	26	28	15	Female
687	1994	26	0	0	14	Female
688	2005	32	24	10	24	Female
689	2005	25	0	1	27	Male
690	2001	51	13	34	22	Female
691	2005	61	45	46	15	Female
692	2003	56	46	31	19	Female
693	2000	25	1	2	5	Female
694	2004	55	25	36	25	Female
695	2004	27	7	11	20	Female
696	2002	40	25	21	24	Female
697	2005	25	2	12	7	Female
698	2004	92	81	66	29	Female
699	2003	29	4	16	13	Male
700	2003	48	17	22	20	Female
701	2003	25	3	1	13	Male
702	2003	61	43	32	16	Female
703	2004	25	0	6	9	Female
704	2004	25	15	8	16	Female
705	2005	33	19	7	20	Female
706	2003	29	7	11	20	Female
707	2004	36	11	25	9	Male
708	2004	26	1	3	16	Male
709	2002	37	32	23	13	Female
710	2005	34	29	40	21	Female
711	2005	32	32	31	19	Female
712	2004	51	18	11	25	Female
713	2002	29	4	4	12	Female

714	2005	32	8	14	15	Male
715	2005	37	65	65	26	Female
716	2005	27	9	4	13	Male
717	2002	25	1	0	12	Female
718	2005	45	23	21	20	Female
719	2005	36	24	10	17	Female
720	2004	63	45	47	16	Female
721	2004	41	18	47	17	Female
722	2005	28	10	5	12	Female
723	2003	28	5	5	8	Female
724	2004	73	61	50	28	Female
725	2004	28	6	7	15	Female
726	2005	25	1	0	10	Female
727	2004	64	43	47	23	Female
728	2005	69	55	42	24	Female
729	2004	41	27	41	26	Male
730	2005	25	0	0	22	Male
731	2004	35	19	31	14	Female
732	2004	33	5	10	17	Female
733	2004	72	67	51	35	Other (please ty
734	2005	25	2	0	19	Male
735	2003	27	6	15	19	Female
736	2005	41	31	24	23	Male
737	2005	35	18	17	16	Female
738	2004	49	23	19	17	Male
739	2003	30	9	9	20	Female
740	2005	26	4	0	17	Female
741	2005	56	60	28	16	Female
742	2004	59	43	32	18	Female
743	2004	41	14	8	18	Female
744	2005	28	0	3	19	Male
745	2005	42	22	28	25	Male
746	2004	25	3	4	10	Female
747	2002	48	38	39	22	Female
748	2004	48	30	33	29	Male
749	2003	25	3	8	26	Female
750	2001	32	28	13	19	Female
751	2003	33	12	8	27	Female
752	2004	27	4	2	19	Female
753	2005	62	58	50	32	Female
754	2003	35	11	15	16	Female
755	2002	51	14	36	16	Female

756	2003	35	24	18	22	Female
757	2005	27	3	0	14	Female
758	2004	27	5	6	24	Female
759	2004	83	82	39	27	Female
760	2005	25	8	10	10	Female
761	2004	25	2	1	21	Female
762	2004	45	22	37	20	Female
763	2005	35	3	22	15	Female
764	2003	73	47	36	29	Male
765	2004	36	14	10	10	Male
766	2004	41	39	34	17	Male
767	2005	35	0	7	21	Female
768	2005	37	30	25	22	Female
769	2004	30	4	5	8	Female
770	2005	55	55	41	29	Female
771	2005	36	5	5	16	Female
772	2005	50	20	31	18	Male
773	2005	34	8	11	14	Male
774	2001	62	35	31	23	Female
775	2003	34	10	10	14	Female
776	2002	62	51	50	26	Female
777	2003	39	31	38	32	Female
778	2005	84	74	48	31	Female
779	2001	55	47	43	23	Female
780	2002	37	12	16	23	Female
781	2002	33	24	38	13	Female
782	2005	30	5	2	20	Female
783	2003	28	4	6	19	Male
784	2005	30	18	7	20	Female
785	2004	25	0	14	15	Male
786	2002	33	14	8	28	Female
787	2004	57	49	38	22	Female
788	2005	25	1	3	19	Female
789	2003	59	52	45	30	Female
790	2005	34	9	12	21	Female
791	2003	27	6	14	16	Female
792	2002	43	5	8	29	Female
793	2003	57	35	32	34	Female
794	2004	65	47	35	17	Female
795	2004	31	4	2	21	Female
796	2004	25	3	4	17	Female
797	1993	26	3	1	24	Male

798	2005	32	13	16	14	Female
799	2005	31	5	2	11	Female
800	2005	25	0	0	18	Female
801	2005	29	11	6	14	Female
802	2004	34	31	30	16	Female
803	2003	72	56	47	43	Female
804	2004	30	9	19	25	Female
805	2005	54	49	38	23	Male
806	2005	31	9	18	26	Male
807	2005	42	16	15	21	Female
808	2005	26	1	0	12	Female
809	2023	36	17	11	16	Female
810	2003	34	16	13	25	Female
811	2005	38	15	25	22	Female
812	2005	25	4	0	16	Female
813	2005	32	23	8	18	Female
814	2003	56	38	37	26	Female
815	2001	53	38	48	19	Female
816	2003	29	7	3	9	Female
817	2005	31	6	0	23	Male
818	2002	37	21	24	26	Female
819	2003	33	12	4	31	Male
820	2002	42	16	20	19	Female
821	2002	27	6	0	10	Female
822	2003	38	38	30	19	Female
823	2002	68	31	35	23	Male
824	2005	71	64	59	27	Female
825	2005	36	11	15	28	Male
826	2005	65	50	48	17	Female
827	2004	28	29	17	15	Female
828	2005	29	41	54	13	Female
829	2005	45	47	39	22	Female
830	2004	35	0	0	18	Male
831	2005	30	5	1	21	Female
832	2005	39	29	12	15	Male
833	2005	64	83	52	23	Female
834	1989	28	0	0	17	Male
835	2002	36	33	22	19	Female
836	2003	37	21	29	22	Female
837	2005	44	16	21	29	Female
838	2005	73	64	37	21	Female
839	2003	25	1	12	22	Female

840	2005	54	21	15	17	Female
841	2005	26	1	0	19	Female
842	2005	45	31	27	12	Female
843	2003	30	7	0	16	Female
844	2003	29	4	2	13	Female
845	2005	59	74	48	16	Female
846	2005	36	16	47	24	Female
847	2005	26	9	7	14	Female
848	2005	27	3	0	14	Male
849	2005	49	37	46	29	Female
850	2005	45	16	31	28	Female
851	2005	25	0	0	11	Female
852	2005	33	3	19	17	Male
853	2002	38	18	32	25	Female
854	2003	37	16	16	22	Male
855	2005	38	41	20	16	Female
856	2005	56	47	38	15	Female
857	2004	25	8	7	20	Female
858	2004	25	4	1	24	Male
859	2004	25	0	0	23	Male
860	2005	38	47	2	18	Male
861	2005	29	0	24	15	Female
862	2005	77	63	64	27	Female
863	2004	51	19	37	22	Female
864	2005	25	7	3	17	Female
865	2023	25	0	0	13	Male
866	2005	38	15	59	20	Female
867	2005	50	19	22	28	Male
868	2005	28	23	5	27	Male
869	2003	25	0	0	18	Female
870	2005	25	0	0	20	Male
871	2005	54	34	13	13	Male
872	1996	35	21	9	15	Female
873	2004	32	9	43	27	Female
874	2000	51	33	58	23	Female
875	1999	61	71	55	25	Female
876	2005	25	2	0	16	Female
877	2005	55	68	15	18	Female
878	2004	25	0	0	21	Female
879	2005	43	17	26	21	Female
880	2005	25	1	0	26	Male
881	2005	25	0	0	21	Female

882	2003	28	17	19	6	Female
883	2003	47	0	14	16	Female
884	2003	48	23	23	27	Female
885	1999	26	0	0	13	Female
886	2005	26	4	13	15	Male
887	2004	25	0	1	22	Female
888	2005	25	0	0	11	Female
889	2005	27	22	7	24	Female
890	2005	32	13	1	13	Female
891	1998	31	3	6	26	Male
892	2004	67	54	42	19	Female
893	2005	28	8	2	17	Female
894	2003	70	70	71	26	Female
895	2003	36	16	12	16	Female
896	2005	29	15	30	25	Male
897	2004	28	0	0	11	Female
898	2005	42	18	11	23	Female
899	2004	25	0	0	16	Male
900	2005	47	49	39	21	Female
901	2002	77	67	44	25	Female
902	2005	73	36	25	26	Female
903	2005	48	24	27	13	Male
904	2004	47	20	35	8	Female
905	2005	59	29	55	24	Female
906	2005	41	41	35	28	Female
907	2005	75	46	41	22	Male
908	2001	28	0	0	14	Female
909	2003	59	29	33	14	Female
910	2005	26	14	2	17	Female
911	2005	51	44	42	21	Female
912	2004	25	6	0	28	Male
913	2003	47	40	28	27	Female
914	2005	24	3	0	22	Female
915	2005	40	33	54	21	Female
916	2003	50	39	20	24	Female
917	2005	25	0	0	19	Male
918	2005	48	31	41	24	Female
919	2004	25	2	0	19	Female
920	2004	39	18	19	24	Female
921	2005	35	4	3	16	Male
922	2005	38	15	29	26	Male
923	2003	86	66	56	39	Prefer not to ans

924	2005	37	24	32	17	Female
925	2004	30	8	16	12	Male
926	2004	62	50	54	17	Female
927	2005	92	92	49	34	Other (please ty
928	2005	25	0	0	17	Female
929	2004	35	8	37	12	Female
930	2004	63	47	40	17	Female
931	2003	29	9	62	19	Male
932	2005	38	33	28	22	Female
933	2002	51	29	70	9	Female
934	2005	25	1	1	32	Female
935	2002	33	24	21	17	Female
936	2005	32	11	8	24	Female
937	2005	73	81	80	26	Female
938	2004	31	27	29	11	Female
939	2003	26	1	0	23	Female
940	2005	27	6	1	17	Female
941	2005	28	11	18	23	Female
942	2005	26	10	26	15	Female
943	2005	73	31	42	35	Male
944	2005	30	11	4	21	Female
945	2004	26	0	0	21	Male
946	2005	29	10	16	25	Female
947	2004	45	42	23	23	Male
948	2002	25	0	10	12	Male
949	2003	28	2	4	18	Male
950	2004	66	52	51	28	Male
951	2004	28	16	1	20	Male
952	2004	46	36	21	20	Female
953	2005	32	12	18	26	Female
954	2005	49	47	41	22	Female
955	2005	31	18	35	18	Female
956	2005	61	53	50	23	Female
957	2002	70	51	33	17	Female
958	2003	42	20	17	13	Male
959	2003	50	48	30	24	Female
960	2005	53	25	50	29	Female
961	2003	36	11	22	20	Female
962		47	31	22	21	Female
963	2004	50	26	35	11	Female
964	2004	46	32	37	19	Female
965	2005	35	26	14	20	Female

966	2002	27	13	13	22	Male
967	2005	30	7	2	18	Female
968	2005	50	27	46	23	Female
969	2005	59	49	42	19	Female
970	2005	70	66	57	30	Female
971	2004	25	8	0	10	Female
972	1999	25	0	0	16	Male
973	2005	25	1	0	18	Female
974	2003	36	27	55	19	Female
975	2002	33	15	31	24	Female
976	2005	40	34	28	14	Female
977	2005	30	3	3	19	Female
978	2004	98	73	68	29	Female
979	2005	72	54	29	32	Female
980	2003	47	15	32	28	Female
981	2004	26	20	18	10	Female
982	2005	37	10	19	12	Female
983	2004	36	32	61	21	Other (please ty
984	2001	56	38	45	23	Male
985	2004	31	11	13	17	Female
986	2004	26	3	5	18	Male
987	2004	36	11	21	21	Female
988	2005	28	54	48	17	Female
989	2005	26	1	9	16	Female
990	2023	32	4	0	16	Male
991	2003	36	7	7	15	Female
992	2004	26	0	0	13	Male
993	2004	26	0	0	18	Male
994	2005	33	17	24	14	Female
995	2004	63	49	22	28	Female
996	2005	39	31	37	29	Female
997	2002	25	1	1	16	Female
998	2023	32	9	1	30	Male
999	2004	32	27	37	18	Male
1000	2002	27	4	11	13	Female
1001	2003	37	44	62	18	Female
1002	2005	43	12	62	18	Male
1003	2001	27	14	12	26	Female
1004	2005	25	1	0	20	Female
1005	2005	26	6	4	14	Female
1006	2003	25	0	0	14	Female
1007	2004	71	69	18	29	Female

1008	2003	37	44	39	21	Prefer not to ans
1009	2003	50	48	39	19	Female
1010	2005	26	2	1	9	Female
1011	2005	50	20	42	28	Male
1012	2005	41	31	40	14	Female
1013	2004	32	6	22	17	Female
1014	2005	44	25	30	23	Female
1015	2005	51	35	37	29	Male
1016	2001	36	42	23	17	Female
1017	2004	36	37	34	19	Female
1018	2023	29	6	16	21	Female
1019	2023	29	8	15	20	Female
1020	2005	30	37	28	15	Female
1021	2005	72	37	56	23	Female
1022	2002	47	33	39	27	Female
1023	2003	54	34	41	16	Female
1024	1996	29	4	10	15	Female
1025	2004	46	12	16	32	Female
1026	2005	25	6	4	10	Female
1027	2005	27	0	9	17	Female
1028	2005	49	38	42	20	Female
1029	2004	32	6	12	21	Female
1030	2005	52	17	33	19	Male
1031	2005	25	34	0	20	Female
1032	2005	29	0	1	15	Male
1033	2003	47	30	23	24	Female
1034	2005	26	1	0	9	Male
1035	2005	36	11	19	19	Female
1036	2005	39	25	32	20	Female
1037	2005	28	3	4	15	Female
1038	2005	25	0	0	14	Female
1039	2005	26	4	10	33	Male
1040	2004	25	23	1	21	Female
1041	2002	75	40	43	23	Male
1042	2004	42	33	30	16	Male
1043	1999	37	6	11	16	Female
1044	2005	25	4	0	12	Female
1045	2001	28	3	4	20	Female
1046	2005	48	35	24	25	Male
1047	2005	29	4	13	16	Female
1048	2004	61	40	43	13	Female
1049	2005	33	20	11	15	Female

1050	2002	58	51	46	18	Female
1051	2003	27	10	21	16	Female
1052	2002	24	9	2	34	Female
1053	2005	30	11	3	23	Male
1054	2004	39	36	28	24	Female
1055	2004	80	19	40	40	Male
1056	2002	24	0	0	11	Female
1057	2005	37	11	11	20	Female
1058	2005	25	0	0	8	Female
1059	2005	31	12	8	22	Female
1060	2001	56	21	17	18	Male
1061	2005	51	28	38	21	Female
1062	2005	32	24	22	21	Female
1063	2005	25	9	4	22	Male
1064	2005	28	8	20	16	Female
1065	1992	56	34	38	30	Female
1066	2005	68	53	23	21	Female
1067	2004	26	19	19	18	Male
1068	1995	28	0	0	10	Male
1069	2003	54	24	17	27	Female
1070	2002	46	56	30	31	Female
1071	2004	25	0	1	11	Female
1072	2005	43	0	0	29	Female
1073	2005	59	49	33	25	Female
1074	2005	25	0	0	14	Male
1075	2005	52	57	70	18	Female
1076	2005	55	40	59	29	Female
1077	2005	80	62	72	12	Female
1078	2003	36	21	21	20	Male
1079	2003	24	6	18	22	Female
1080	2004	30	4	2	21	Male
1081	2005	25	0	0	12	Male
1082	2001	47	40	40	21	Male
1083	2004	36	11	30	14	Male
1084	2004	27	2	2	12	Female
1085	2005	35	13	17	33	Female
1086	2004	37	32	22	21	Female
1087	2002	25	0	0	13	Female
1088	2005	43	42	30	24	Female
1089	2003	26	0	0	30	Male
1090	2005	56	43	44	23	Female
1091	2003	86	68	55	45	Female

1092	2003	43	39	59	27	Female
1093	2005	25	0	0	16	Female
1094	2004	75	42	29	24	Male
1095	2002	41	23	12	15	Female
1096	2003	25	0	0	20	Male
1097	2002	42	5	39	22	Female
1098	2005	36	10	0	22	Male
1099	2005	72	36	42	25	Female
1100	2005	26	0	1	17	Male
1101	2005	30	6	12	28	Male
1102	2003	29	7	11	12	Male
1103	2005	26	0	0	10	Female
1104	2005	25	27	14	9	Female
1105	2005	42	20	33	21	Female
1106	2005	25	1	0	7	Female
1107	2004	70	61	49	30	Female
1108	2005	53	60	47	34	Female
1109	2005	89	51	49	29	Female
1110	2000	78	65	47	41	Female
1111	2004	44	36	23	19	Female
1112	2003	37	41	37	19	Female
1113	2005	27	34	37	24	Female
1114	2005	29	21	4	18	Male
1115	2004	65	61	28	43	Female
1116	2003	69	59	49	29	Female
1117	2004	25	7	20	15	Male
1118	2004	26	2	0	13	Male
1119	2004	33	13	22	30	Female
1120	2005	26	5	5	9	Female
1121	2002	28	13	6	16	Female
1122	2004	37	27	7	13	Female
1123	2004	50	45	51	15	Female
1124		27	0	5	14	Female
1125	2002	41	25	20	28	Female
1126	2003	63	53	45	29	Female
1127	2003	38	33	12	27	Female
1128	2003	63	53	37	34	Female
1129	2005	67	58	56	17	Female
1130	2003	26	0	2	12	Female
1131	2003	28	14	7	14	Female
1132	2004	46	30	51	15	Female
1133	2000	25	1	1	22	Male

1134	2004	40	8	24	24	Female
1135	2004	27	8	0	23	Female
1136	2000	52	28	12	22	Female
1137	2006	25	0	0	25	Male
1138	2004	69	66	37	20	Female
1139	1997	50	24	11	30	Female
1140	2003	72	65	26	35	Female
1141	2003	32	8	14	20	Female
1142	2005	29	5	6	17	Female
1143	2002	44	42	17	15	Female
1144	2004	44	14	26	22	Male
1145	2004	32	4	9	25	Female
1146	2005	27	0	0	15	Female
1147	2001	46	34	10	17	Female
1148	2005	40	44	52	24	Female
1149	2004	35	20	10	13	Female
1150	2004	25	0	0	15	Female
1151	2005	31	9	11	19	Male
1152	2005	25	6	1	23	Male
1153	2004	29	5	19	18	Female
1154	2002	25	0	0	21	Male
1155	2004	29	0	0	24	Male
1156	2003	37	14	14	23	Female
1157	2004	25	1	3	31	Female
1158	2005	69	36	42	26	Female
1159	2003	53	21	29	28	Female
1160	2004	35	17	18	35	Female
1161	2005	30	14	13	23	Female
1162	2002	25	1	1	12	Female
1163	2004	50	40	40	22	Other (please ty
1164	2004	44	41	59	18	Male
1165	2001	28	3	3	18	Male
1166	2003	36	12	13	19	Male
1167	2004	28	7	0	13	Female
1168	2004	79	74	66	26	Female
1169	2002	29	4	8	18	Female
1170	2005	63	61	38	19	Female
1171	2003	25	2	3	28	Male
1172	2003	44	38	53	16	Female
1173	2005	30	2	0	22	Male
1174	2005	27	12	8	20	Female
1175	2003	37	8	7	18	Male

1176	2004	48	49	37	16	Female
1177	2000	32	4	16	12	Female
1178	2002	27	0	0	19	Male
1179	2004	31	6	19	6	Male
1180	2005	37	7	17	24	Female
1181	2005	37	20	17	23	Other (please ty
1182	2005	52	57	63	10	Female
1183	2023	33	3	27	11	Male
1184	2005	38	27	23	11	Female
1185		25	4	8	15	Male
1186	2005	27	8	3	14	Male
1187	2004	25	3	2	15	Female
1188	2001	38	31	24	29	Male
1189	2003	27	7	5	11	Female
1190	2005	71	45	36	21	Female
1191	2005	40	17	13	20	Female
1192	2005	33	13	34	24	Male
1193	2004	45	16	29	16	Male
1194	2005	30	6	13	16	Female
1195	2005	33	10	1	15	Male
1196	2000	25	1	2	10	Female
1197	2003	25	0	0	12	Female
1198	2005	30	11	21	18	Male
1199	2003	41	23	6	13	Female
1200	2005	54	36	26	34	Female
1201	2005	25	0	18	14	Female
1202	2004	36	21	48	26	Female
1203	2004	88	50	73	26	Male
1204	2004	36	37	39	31	Female
1205	2002	30	4	0	22	Male
1206	1999	23	28	17	27	Female
1207	2004	54	37	43	19	Male
1208	2003	92	83	57	24	Female
1209	2005	25	1	10	16	Male
1210	2005	29	0	0	22	Female
1211	2005	36	10	4	12	Male
1212	2001	49	22	35	27	Female
1213	2005	32	12	10	18	Female
1214	2003	28	5	16	18	Male
1215	2004	25	1	8	11	Male
1216	2005	37	15	9	23	Male
1217	2005	25	0	0	18	Male

1218	2005	27	0	0	11	Male
1219	2023	36	16	20	17	Male
1220	2005	40	21	25	23	Female
1221	2001	35	5	5	8	Female
1222	2002	33	7	7	19	Female
1223	2002	31	25	37	20	Female
1224	2005	29	27	27	17	Female
1225	2002	49	38	27	16	Female
1226	2002	26	6	16	15	Female
1227	2003	70	64	48	26	Female
1228	2002	48	0	0	33	Male
1229	2005	30	9	4	13	Female
1230	2003	28	2	7	14	Female
1231	2002	26	4	15	19	Female
1232	2004	37	11	7	28	Female
1233	2005	46	28	42	21	Female
1234	2004	40	35	26	19	Male
1235	2002	25	0	0	7	Female
1236	1999	79	48	48	28	Female
1237	2004	50	52	58	14	Female
1238	2003	30	17	29	34	Female
1239	2002	37	30	24	17	Female
1240	2005	56	24	29	21	Female
1241		27	1	7	23	Female
1242	2002	55	65	39	17	Female
1243	2001	25	0	0	22	Male
1244	2005	28	13	8	16	Male
1245	2005	26	6	2	32	Female
1246	2005	29	4	10	21	Female
1247	2002	24	15	0	12	Male
1248	2002	41	34	32	20	Female
1249	2004	59	48	33	23	Female
1250	2004	25	0	0	11	Male
1251	2004	52	51	42	28	Female
1252	2005	39	18	6	17	Female
1253	2002	45	22	25	27	Male
1254	2005	25	3	8	16	Female
1255	2004	25	0	16	16	Male
1256	2005	48	48	28	13	Female
1257	2004	38	27	28	17	Female
1258	2005	32	9	5	17	Female
1259	2002	27	1	11	14	Female

1260	2004	54	34	20	19	Female
1261	2004	25	16	0	17	Male
1262	2004	49	41	34	21	Female
1263	2003	37	16	6	16	Female
1264	2004	26	10	22	23	Female
1265	2005	57	37	24	18	Female
1266	2002	41	14	0	18	Female
1267	2001	48	12	19	32	Female
1268	2003	25	0	0	21	Male
1269	2001	27	6	10	22	Female
1270	2003	26	14	18	27	Female
1271	2005	39	33	23	27	Female
1272	2003	27	5	6	16	Female
1273	2002	64	48	31	22	Male
1274	1991	37	5	5	14	Female
1275	2005	25	0	0	11	Female
1276	2001	32	15	34	18	Female
1277	2004	33	12	17	17	Female
1278	2005	25	0	0	24	Female
1279	2002	33	0	13	17	Female
1280	2003	60	28	67	29	Prefer not to an:
1281	2001	37	20	19	26	Female
1282	2002	29	3	2	14	Female
1283	2005	62	43	44	18	Female
1284	2004	25	0	0	24	Female
1285	1991	43	27	28	15	Transgender
1286	2004	46	27	28	21	Female
1287	2005	28	0	9	16	Female
1288	2000	25	14	10	16	Female
1289	2023	26	3	1	26	Female
1290	2004	27	5	3	30	Female
1291	2005	25	0	0	18	Male
1292	2005	27	0	0	14	Male
1293	2023	54	60	53	23	Female
1294	2004	34	20	22	21	Male
1295	2004	43	25	34	18	Female
1296	2002	25	14	17	32	Female
1297	2023	25	2	1	17	Female
1298	2002	26	12	9	31	Female
1299	2003	25	0	7	9	Male
1300	2004	35	10	12	16	Female
1301	2004	34	56	38	14	Female

1302	2004	26	9	9	22	Female
1303	2005	25	0	0	22	Male
1304	1989	29	3	6	20	Male
1305	2005	25	8	3	24	Female
1306	2005	40	24	14	24	Female
1307	2003	48	30	43	22	Female
1308	2003	34	13	26	18	Female
1309	2002	55	41	17	27	Female
1310	2003	47	47	43	16	Other (please ty
1311	2002	26	14	2	20	Female
1312	2022	56	77	48	24	Male
1313	2003	28	1	8	23	Male
1314	2005	58	59	43	18	Male
1315	2003	33	4	4	14	Female
1316	2004	40	16	47	19	Female
1317	2005	64	39	42	32	Male
1318	2003	25	1	3	9	Male
1319	2003	32	19	12	18	Female
1320	2002	39	20	34	17	Female
1321	2005	28	4	12	34	Male
1322	2001	66	25	27	32	Male
1323	2005	29	8	12	9	Female
1324	2004	60	56	48	19	Female
1325	2004	52	27	38	14	Female
1326	2002	27	5	4	14	Female
1327	2004	71	41	47	26	Female
1328	2005	24	3	0	20	Female
1329	2002	50	46	32	19	Female
1330	2005	28	7	8	22	Female
1331	2001	36	9	15	16	Female
1332	2004	30	10	10	11	Male
1333	2003	58	21	41	30	Female
1334	2004	35	29	11	13	Female
1335	1999	51	4	3	16	Male
1336	2004	28	8	31	23	Female
1337	2005	34	16	16	15	Female
1338	2004	33	20	21	28	Female
1339		36	28	36	19	Female
1340	2005	25	15	0	16	Female
1341	2002	40	4	2	21	Female
1342	2005	28	2	2	10	Female
1343	2002	30	6	11	23	Female

1344	2003	45	44	44	32	Female
1345	2005	25	0	0	26	Male
1346	1999	25	8	7	25	Male
1347	2004	74	76	55	29	Female
1348	2000	51	42	21	24	Male
1349	2002	60	20	24	23	Female
1350	2003	35	42	22	33	Female
1351	2002	24	2	0	16	Male
1352	2005	53	13	28	20	Male
1353	2004	64	33	29	22	Female
1354	2005	52	44	27	16	Female
1355	2004	32	52	43	23	Female
1356		68	35	36	30	Female
1357	2002	81	81	67	30	Prefer not to ans
1358	2000	37	12	16	26	Male
1359	2005	29	2	4	9	Male
1360	2004	39	24	40	17	Female
1361	2003	25	0	0	14	Female
1362	2005	29	8	10	19	Male
1363	2003	36	10	18	30	Male
1364	2003	46	48	31	27	Female
1365	2002	25	14	6	25	Female
1366	2005	31	35	13	18	Female
1367	2005	35	9	3	10	Male
1368	1995	41	12	4	18	Male
1369	2001	34	10	11	15	Male
1370	1999	58	31	37	30	Female
1371	2002	24	2	0	12	Female
1372	2003	39	29	23	13	Female
1373	2004	73	62	40	25	Female
1374	2003	74	55	45	16	Female
1375	2004	38	5	28	19	Male
1376	1998	26	1	5	15	Male
1377	2005	30	0	0	16	Female
1378	2005	27	3	3	13	Female
1379	2002	59	33	37	23	Female
1380	2005	26	1	4	14	Female
1381	2005	34	9	32	24	Male
1382	2002	67	60	51	15	Female
1383	2005	28	2	0	13	Male
1384	2003	49	65	24	25	Male
1385	1999	28	12	30	21	Female

1386	2003	58	24	45	32	Female
1387	1995	40	19	35	18	Male
1388	2003	31	0	0	21	Female
1389	2004	26	5	25	11	Female
1390	2004	27	1	4	11	Female
1391	2003	33	8	8	18	Female
1392	2005	52	40	32	23	Female
1393	2002	32	0	4	13	Female
1394	2002	29	7	25	20	Male
1395	2005	52	39	27	19	Female
1396	2005	63	41	33	28	Male
1397	2003	35	3	13	23	Female
1398	2005	32	17	27	16	Female
1399	2003	60	49	26	33	Female
1400	1999	30	12	31	13	Female
1401	2005	28	3	9	18	Female
1402	2004	35	18	16	13	Female
1403	2003	27	1	5	20	Female
1404	2004	42	26	11	25	Male
1405	2005	33	6	22	17	Female
1406	2005	59	19	50	24	Female
1407	2005	55	15	51	19	Female
1408	2005	55	23	64	25	Prefer not to ans
1409	2005	27	9	21	24	Female
1410	2005	75	42	49	32	Female
1411	2002	38	30	26	21	Male
1412	2005	31	5	1	22	Female
1413	2005	25	8	6	18	Female
1414	2004	25	0	0	8	Female
1415	2001	28	10	26	19	Female
1416	2001	33	23	25	33	Male
1417	2004	39	36	1	31	Male
1418	2005	33	12	10	18	Male
1419	2023	50	16	11	32	Female
1420	2005	68	73	55	15	Female
1421	2005	25	12	0	13	Female
1422	2004	39	23	35	23	Female
1423	2004	29	15	22	11	Female
1424	2005	36	33	35	16	Female
1425	2003	69	47	24	19	Female
1426	2004	27	10	5	18	Female
1427	2004	34	8	7	20	Female

1428	2003	37	33	39	34	Female
1429	1986	25	0	0	21	Male
1430	1999	29	3	11	20	Female
1431	2002	61	42	45	29	Female
1432	2005	49	34	38	15	Female
1433	2005	59	40	45	22	Female
1434	2003	37	0	3	16	Female
1435	2004	53	40	29	18	Female
1436	2002	61	37	44	18	Female
1437	2005	26	4	3	22	Female
1438	2003	39	11	22	29	Female
1439	2002	30	6	37	10	Female
1440	1997	34	14	17	23	Female
1441	2003	31	7	7	5	Female
1442	2004	45	30	37	24	Female
1443	2004	61	48	47	32	Other (please ty
1444	2003	33	15	19	16	Female
1445	2003	30	8	11	14	Female
1446	2005	55	33	30	19	Female
1447	2003	51	43	45	24	Female
1448	2003	49	36	41	32	Female
1449	2004	49	26	35	20	Female
1450	2003	32	12	3	17	Female
1451	2005	27	3	12	21	Female
1452	2005	27	29	40	32	Female
1453	1998	58	47	49	27	Female
1454	2003	25	5	0	29	Female
1455	2004	28	2	18	23	Female
1456	2005	33	12	8	19	Male
1457	2000	59	36	29	32	Female
1458	2005	61	49	41	20	Female
1459	1999	62	31	29	32	Transgender
1460	2005	62	29	42	22	Male
1461	2002	66	47	44	12	Female
1462	2005	32	24	24	12	Female
1463	2005	25	13	34	14	Female
1464	2005	65	40	50	18	Female
1465	2005	56	57	30	30	Female
1466	2003	77	31	48	19	Male
1467	2003	31	10	2	29	Female
1468	2005	49	43	55	31	Female
1469	1982	37	20	12	13	Female

1470	2004	25	1	4	21	Female
1471	2005	28	13	37	16	Female
1472	2005	71	71	47	27	Female
1473	2002	33	7	9	24	Male
1474	2005	26	1	0	19	Male
1475	2005	33	14	18	21	Female
1476	2003	34	24	22	18	Female
1477	2001	47	19	40	15	Male
1478	1997	29	14	0	15	Female
1479	2004	28	2	2	16	Male
1480	2002	30	10	9	25	Female
1481	2002	25	0	11	23	Male
1482	2002	59	38	31	27	Female
1483	2005	59	57	46	18	Female
1484	2005	25	1	0	12	Female
1485	2005	60	36	41	27	Female
1486	2005	52	27	70	12	Female
1487	2005	55	35	46	12	Female
1488	2002	25	1	3	10	Female
1489	2002	37	25	13	18	Female
1490	1999	32	34	28	21	Male
1491	2005	38	12	39	13	Female
1492	2003	30	4	3	14	Female
1493	2005	30	23	18	15	Female
1494	2003	25	8	15	13	Female
1495	2003	69	35	53	20	Female
1496	2004	31	3	11	25	Female
1497	2003	25	9	6	13	Female
1498	1984	54	33	28	28	Female
1499	1991	29	5	20	26	Female
1500	1997	31	11	1	28	Female
1501	2005	25	0	0	24	Female
1502	2004	26	0	1	12	Female
1503	2003	70	55	41	29	Female
1504	2003	32	29	6	16	Female
1505	2003	32	19	8	27	Female
1506	2005	33	11	25	13	Female
1507	2005	25	2	1	9	Female
1508	2002	33	21	18	18	Female
1509	2005	24	8	6	27	Male
1510	2001	30	18	18	12	Female
1511	2005	26	7	11	13	Female

1512	2000	54	60	23	16	Female
1513	2003	25	22	3	12	Female
1514	2005	43	5	0	10	Female
1515	2004	42	15	19	18	Male
1516	2002	56	28	34	23	Female
1517	2003	35	27	21	16	Female
1518	2003	25	0	0	17	Female
1519	2003	31	11	16	14	Female
1520	2004	47	37	40	15	Female
1521	2003	33	16	16	15	Female
1522	2004	66	45	40	19	Female
1523	2004	65	34	36	28	Female
1524	2004	41	31	20	29	Female
1525	2003	29	0	0	20	Female
1526	2003	26	8	7	18	Female
1527	2005	30	5	4	13	Female
1528	2004	33	36	24	14	Female
1529	1997	64	59	49	18	Female
1530	2005	25	0	0	17	Female
1531	2005	31	4	9	27	Female
1532	2004	32	5	3	14	Male
1533	2000	26	4	6	9	Female
1534	2005	39	16	10	22	Female
1535	2004	71	47	46	41	Female
1536	2005	31	6	0	22	Female
1537	2003	31	10	12	23	Female
1538	2005	50	52	42	26	Female
1539	2005	34	29	21	12	Female
1540	2004	28	0	6	16	Male
1541	2005	37	26	26	18	Female
1542	2004	58	35	47	20	Female
1543	2001	37	17	6	18	Female
1544	2005	47	21	23	16	Female
1545	2005	93	74	60	29	Other (please ty
1546	2005	33	0	0	23	Female
1547	2004	26	0	0	16	Male
1548	2005	28	8	0	30	Male
1549	2004	32	14	7	16	Female
1550	2004	29	1	3	21	Male
1551	2004	25	10	3	23	Female
1552	2001	60	59	43	27	Female
1553	2005	46	33	26	22	Female

1554	2005	65	48	54	25	Female
1555	2004	31	17	15	17	Female
1556	2005	28	18	4	28	Female
1557	2004	25	4	5	17	Female
1558	2004	51	20	26	41	Female
1559	2003	34	23	47	16	Female
1560	2002	44	36	42	15	Female
1561	2002	25	21	1	23	Male
1562	2005	50	33	42	20	Female
1563	2005	46	22	15	24	Female
1564	2005	58	39	53	29	Female
1565	2005	52	36	39	18	Female
1566	2005	40	39	24	25	Female
1567	2004	53	39	31	25	Female
1568	2001	31	9	12	14	Female
1569	2005	54	29	26	28	Other (please ty
1570	2004	42	36	25	28	Female
1571	2004	43	35	32	34	Female
1572	2005	27	6	0	12	Male
1573	2004	40	34	60	18	Female
1574	2005	33	12	4	25	Female
1575	2005	26	12	25	22	Female
1576	2002	31	0	0	13	Female
1577	2004	47	28	26	23	Female
1578	2002	52	61	31	15	Female
1579	2003	25	2	4	31	Male
1580	2002	41	20	10	23	Female
1581	2005	25	11	10	9	Female
1582	2005	32	1	21	11	Female
1583	2005	30	6	1	20	Female
1584	2005	26	7	2	13	Female
1585	2005	32	30	32	23	Female
1586	2005	30	7	39	17	Male
1587	2004	39	11	21	25	Female
1588	2002	34	7	24	34	Female
1589	2000	49	42	40	31	Female
1590	2003	33	0	0	19	Female
1591	2005	62	49	31	23	Female
1592	2005	33	3	7	15	Female
1593	2004	42	36	34	21	Female
1594	2002	52	34	36	27	Male
1595	2004	51	33	35	25	Female

1596	1987	34	30	19	6	Female
1597	2003	47	33	48	22	Female
1598	2004	51	26	13	16	Female
1599	2003	27	7	6	12	Female
1600	2003	100	52	27	33	Transgender
1601	2005	26	3	3	13	Female
1602	2005	25	8	15	32	Male
1603	2003	42	31	10	38	Female
1604	1999	34	8	4	19	Male
1605	2005	25	0	10	13	Female
1606	2005	46	57	22	27	Female
1607	1988	26	0	5	26	Female
1608	2002	46	26	4	13	Female
1609	2005	26	14	15	18	Female
1610	2003	32	11	27	35	Female
1611	2004	33	17	14	16	Female
1612	2004	34	60	32	32	Female
1613	2005	32	1	3	16	Female
1614	2005	28	2	1	11	Female
1615	2002	26	22	2	16	Female
1616	2005	37	15	14	15	Female
1617	2005	47	31	42	24	Female
1618	2004	69	36	48	18	Other (please ty
1619	2005	25	3	5	9	Male
1620	2003	56	46	39	18	Female
1621	2005	28	2	0	14	Female
1622	2005	33	2	2	20	Male
1623	2002	62	40	47	23	Female
1624	2005	61	23	20	28	Female
1625	2004	25	3	3	19	Female
1626	2005	26	9	17	12	Female
1627	2001	25	41	17	25	Female
1628	2005	28	2	0	23	Female
1629	2005	29	25	21	22	Female
1630	2005	31	10	19	14	Female
1631	2003	31	11	19	16	Female
1632	2002	25	5	0	10	Female
1633	2004	49	21	22	22	Female
1634	2003	48	17	13	28	Female
1635	2005	38	6	27	22	Female
1636	2005	26	1	4	20	Female
1637	2004	30	4	14	26	Male

1638	2003	29	8	2	20	Female
1639	2005	85	86	61	37	Other (please ty
1640	2004	28	12	6	22	Female
1641	2005	35	11	40	19	Female
1642	2002	65	37	33	24	Female
1643	2004	30	0	4	21	Female
1644	2004	26	2	0	19	Female
1645	2002	65	45	44	25	Female
1646	2004	32	12	4	17	Female
1647	2000	32	9	29	19	Male
1648	2005	57	39	26	27	Female
1649	2005	44	33	22	26	Female
1650	2005	25	5	0	27	Female
1651	2000	45	28	37	18	Female
1652	2005	38	6	7	14	Female
1653	2002	76	63	48	20	Female
1654	2002	35	12	17	10	Female
1655	2004	27	1	2	13	Female
1656	2003	25	0	0	25	Male
1657	2002	25	4	6	25	Male
1658	2006	40	46	47	20	Female
1659	2005	39	34	32	16	Female
1660	2002	80	55	63	37	Female
1661	2003	38	9	15	28	Male
1662	2002	32	9	2	9	Male
1663	2002	31	12	5	17	Female
1664	2004	48	38	30	31	Female
1665	2002	49	1	32	19	Female
1666	2005	25	25	17	15	Female
1667	2002	41	13	28	27	Female
1668	2004	25	0	0	18	Female
1669	2004	40	23	27	18	Female
1670	2002	31	2	31	18	Female
1671	2005	49	15	53	12	Female
1672	2002	46	22	24	20	Female
1673	2001	44	28	5	16	Female
1674	2005	78	67	64	21	Female
1675	2005	59	38	33	26	Female
1676	2003	31	10	8	15	Female
1677	2004	36	24	36	21	Female
1678	2003	39	25	34	14	Female
1679	2005	60	44	33	26	Female

1680	2004	79	79	48	29	Female
1681	2003	55	26	38	16	Female
1682	2005	49	51	30	15	Female
1683	2004	33	12	11	14	Male
1684	2003	40	23	33	14	Female
1685	2005	25	0	0	19	Prefer not to ans
1686	2003	27	1	0	12	Female
1687	2005	25	3	2	14	Female
1688	2004	67	27	20	30	Male
1689	2002	60	55	50	19	Female
1690	2005	28	7	11	16	Female
1691	2003	26	3	12	20	Female
1692	2002	62	56	20	23	Female
1693	2005	30	22	33	15	Female
1694	1999	44	6	37	25	Male
1695	2005	37	4	15	21	Female
1696	2005	32	10	23	26	Female
1697	2005	61	33	33	15	Male
1698	2005	60	52	32	17	Female
1699	2002	30	8	12	11	Female
1700	2004	40	5	12	10	Female
1701	2005	26	16	11	19	Female
1702	2002	25	5	0	19	Female
1703	2005	29	6	2	11	Female
1704	2005	25	2	0	28	Female
1705	2003	71	55	51	21	Female
1706	2004	53	48	38	26	Transgender
1707	2004	25	1	0	15	Female
1708	2004	75	63	63	27	Female
1709	2004	75	59	42	34	Other (please ty
1710	1978	50	42	28	17	Female
1711	1999	25	2	32	15	Female
1712	1993	25	0	0	22	Male
1713	2004	35	31	23	31	Female
1714	2005	26	10	12	20	Male
1715	2005	30	33	39	21	Female
1716	2004	37	23	18	24	Female
1717	2002	34	18	4	17	Female
1718	2001	26	4	1	14	Male
1719	2004	54	37	18	29	Female
1720	2003	73	58	49	28	Female
1721	2005	26	14	5	18	Female

1722	2005	27	6	6	18	Female
1723	2005	34	13	9	17	Female
1724	2001	52	24	40	15	Female
1725	1997	44	25	43	22	Male
1726	1979	52	22	27	37	Female
1727	1996	27	7	1	17	Female
1728	2005	43	14	33	15	Male
1729	2004	44	33	22	16	Female
1730	2005	42	19	22	16	Female
1731	2003	31	11	8	10	Female
1732	2004	42	29	23	23	Female
1733	2004	71	60	50	20	Female
1734	2005	35	2	10	20	Female
1735	2005	28	1	0	16	Female
1736	2002	32	20	36	17	Female
1737	2003	48	47	31	27	Other (please ty
1738	2003	44	61	49	14	Male
1739	2005	68	66	46	28	Female
1740	2002	29	13	7	24	Female
1741	2004	25	0	1	28	Female
1742	2003	27	0	5	13	Male
1743	2002	34	24	15	21	Female
1744	2003	40	23	17	20	Female
1745	2003	59	36	31	26	Other (please ty
1746	2001	29	15	16	13	Female
1747	2003	27	0	0	15	Female
1748	2004	31	7	6	17	Female
1749	2005	43	37	16	32	Female
1750	2005	37	8	7	28	Female
1751	2005	50	23	7	19	Female
1752	2002	71	58	40	24	Female
1753	2004	44	35	32	15	Female
1754	2000	25	0	8	20	Female
1755	2003	37	24	14	32	Female
1756	2005	34	31	18	29	Female
1757	2005	33	16	17	16	Female
1758	2004	33	6	9	25	Female
1759	2001	30	8	9	11	Male
1760	2005	28	0	2	13	Female
1761	2002	60	47	37	22	Male
1762	2002	36	24	0	19	Male
1763	2003	32	11	12	20	Male

1764	2004	32	8	19	25	Female
1765	2000	42	35	34	14	Female
1766	2004	28	1	1	13	Female
1767	2003	31	5	7	17	Female
1768	2005	25	0	0	7	Female
1769		47	21	22	25	Female
1770	2003	42	42	26	17	Other (please ty
1771	2003	48	39	37	20	Female
1772	2005	91	72	43	44	Female
1773	2004	54	28	28	31	Female
1774	2004	30	13	13	18	Female
1775	2003	28	1	8	30	Female
1776	2001	30	6	10	5	Female
1777	2004	50	31	30	22	Female
1778	2002	26	16	8	10	Female
1779	2003	39	55	39	24	Male
1780	2004	37	13	19	21	Female
1781	2005	29	17	10	14	Female
1782	2003	30	17	17	16	Female
1783	2001	31	6	11	7	Female
1784	2004	56	30	51	23	Female
1785	2003	28	14	6	9	Female
1786	2005	68	55	60	29	Female
1787	2000	64	51	51	31	Female
1788	2003	33	47	12	16	Female
1789	2004	30	2	5	20	Female
1790	1991	25	0	0	28	Male
1791	2001	48	12	7	29	Female
1792	2000	25	3	1	20	Female
1793	2001	25	0	10	5	Female
1794	2002	26	2	0	12	Male
1795	2004	34	30	16	23	Female
1796		60	38	37	37	Other (please ty
1797	2005	32	10	26	10	Male
1798	2000	35	15	7	22	Female
1799	2002	90	69	55	30	Female
1800	2001	25	0	0	23	Female
1801	2005	44	37	41	32	Female
1802	2005	31	5	13	8	Male
1803	2003	26	2	3	21	Female
1804	2000	41	22	22	21	Female
1805	2004	32	13	30	17	Female

1806	2002	29	9	9	24	Female
1807	2005	25	5	16	12	Female
1808	2003	51	30	29	37	Female
1809	2005	25	0	2	13	Female
1810	2004	25	0	0	20	Female
1811	1999	51	0	0	29	Female
1812	2002	25	0	0	22	Female
1813	2001	58	45	29	26	Female
1814	2005	51	23	30	21	Female
1815	2004	35	13	13	23	Female
1816	2001	29	5	22	20	Female
1817	2002	32	5	17	16	Male
1818	2004	36	7	19	25	Female
1819	2005	25	1	4	14	Female
1820	2002	25	0	0	4	Female
1821	2002	28	1	12	30	Female
1822	2004	31	13	15	19	Female
1823	2002	66	55	39	27	Female
1824	2005	64	67	48	13	Female
1825	2005	31	0	0	17	Female
1826	2003	50	29	25	24	Female
1827	2001	40	33	23	21	Female
1828	2002	25	1	0	15	Male
1829	2003	32	3	8	11	Female
1830	2004	33	8	0	18	Female
1831	2003	35	4	2	24	Female
1832	1997	81	76	48	38	Female
1833	2004	33	13	20	14	Female
1834	2003	26	7	4	15	Female
1835	2001	31	5	5	20	Male
1836	2002	74	59	62	27	Female
1837	2004	52	61	41	14	Female
1838	2003	40	28	20	15	Female
1839	2003	25	0	0	24	Male
1840	2002	49	30	28	19	Female
1841	2004	26	7	18	25	Female
1842	2003	27	21	3	17	Female
1843	2005	47	21	24	22	Male
1844	2005	31	9	7	22	Female
1845	2002	36	31	33	24	Female
1846	2002	37	32	21	20	Female
1847	2004	25	2	0	19	Female

1848	2005	26	1	5	12	Male
1849	2003	45	16	39	34	Male
1850	2003	84	63	53	20	Female
1851	2002	37	11	24	16	Female
1852	2005	25	0	4	21	Female
1853	2005	26	7	11	17	Female
1854	2005	27	4	27	15	Female
1855	2005	31	5	7	23	Female
1856	2002	34	5	21	18	Male
1857	2005	26	0	0	20	Female
1858	2005	47	27	16	22	Female
1859	2005	99	86	45	35	Female
1860	2003	77	53	33	29	Female
1861	2002	37	26	35	25	Male
1862	2003	51	31	23	20	Male
1863	2001	33	0	8	21	Male
1864	1999	46	24	13	22	Female
1865	2003	57	38	45	29	Female
1866	1995	54	16	21	23	Female
1867	2005	50	42	42	30	Female
1868	1995	55	55	55	16	Female
1869	2003	29	3	1	16	Female
1870	2004	27	5	1	11	Female
1871	2004	53	18	34	18	Male
1872	2004	53	30	28	17	Female
1873	2004	29	14	17	12	Female
1874	2005	38	40	57	17	Female
1875	2003	35	11	2	20	Male
1876	2001	29	6	1	18	Female
1877	2003	36	29	21	24	Female
1878	2003	27	8	5	17	Female
1879	2002	25	4	24	16	Female
1880	2005	42	46	29	30	Female
1881	2003	30	3	17	20	Male
1882	1984	59	0	4	19	Male
1883	1999	55	91	84	19	Female
1884	2002	25	0	0	10	Female
1885	2000	69	53	32	19	Female
1886	2004	38	8	26	26	Male
1887		25	8	8	19	Female
1888	2004	53	31	58	15	Female
1889	2003	69	74	38	34	Female

1890	2003	66	41	41	29	Female
1891	2005	25	35	53	28	Female
1892	1999	43	38	50	20	Male
1893	2005	32	1	5	35	Male
1894	1999	35	23	21	16	Female
1895	2004	28	2	0	19	Female
1896	2004	67	43	35	29	Female
1897	2004	31	19	12	19	Female
1898	2004	44	53	59	14	Female
1899	2003	31	22	5	23	Female
1900	2003	62	68	33	34	Female
1901	2004	29	5	0	2	Female
1902		31	17	21	27	Male
1903	2005	30	5	0	13	Female
1904	2003	29	6	13	15	Male
1905	2001	62	30	25	28	Male
1906	2002	31	4	15	15	Female
1907	2002	47	26	25	31	Female
1908	2004	38	11	24	22	Female
1909	2003	37	16	16	21	Male
1910	2002	30	6	2	29	Male
1911	2005	37	17	18	13	Female
1912	2023	48	51	6	22	Female
1913	2001	72	62	61	33	Female
1914	2004	73	44	41	21	Female
1915	2005	29	10	6	19	Female
1916	2005	29	3	8	25	Male
1917	2004	28	12	6	12	Female
1918	2005	41	45	37	17	Female
1919	2005	68	54	47	20	Female
1920	2001	45	39	16	21	Female
1921	2004	62	41	32	23	Female
1922	2005	39	34	36	15	Female
1923	2005	34	10	16	11	Female
1924	2002	31	20	9	21	Female
1925	2003	25	11	0	9	Female
1926	2002	25	8	25	19	Female
1927	2001	47	11	28	23	Female
1928	2004	25	0	4	17	Male
1929	2002	71	59	46	20	Female
1930	2004	64	58	56	34	Female
1931	2002	69	54	46	34	Transgender

1932	1999	28	0	0	21	Female
1933	2002	52	39	11	21	Female
1934	2002	37	12	6	25	Female
1935	2005	32	3	3	13	Female
1936	2004	32	17	44	22	Male
1937	2005	27	3	15	15	Male
1938	2004	25	4	0	12	Female
1939	2001	25	0	12	20	Male
1940	2004	25	5	1	15	Male
1941	2004	33	8	4	24	Female
1942	2005	29	10	11	27	Male
1943	2002	37	9	20	14	Male
1944	2001	48	48	29	26	Male
1945	1995	33	16	34	19	Male
1946	1993	42	7	68	17	Male
1947	2003	46	38	41	20	Female
1948	2002	38	21	6	25	Female
1949	2004	36	2	0	25	Male
1950	2001	25	2	0	15	Male
1951	2005	34	28	16	18	Female
1952	2005	54	26	22	24	Female
1953	2003	28	1	15	18	Female
1954	2005	52	42	46	27	Female
1955	2004	25	0	0	27	Male
1956	1998	28	0	0	6	Male
1957	2005	51	39	34	24	Female
1958	2005	36	9	15	12	Female
1959	2004	43	22	9	26	Female
1960	2005	31	0	2	27	Male
1961	2005	75	60	71	18	Female
1962	2005	25	2	6	21	Female
1963	1998	71	35	46	14	Female
1964	2004	27	6	21	20	Female
1965	2003	25	3	2	11	Male
1966	2002	31	12	15	11	Female
1967	2004	23	1	0	18	Male
1968	2003	38	12	15	13	Female
1969	2004	34	32	17	25	Female
1970	2005	58	51	59	15	Female
1971	2004	30	24	10	15	Female
1972	2004	28	5	0	19	Other (please ty
1973	2004	30	6	9	18	Male

1974	2003	47	0	0	19	Female
1975	2002	62	54	38	22	Other (please ty
1976	2004	27	15	8	17	Male
1977	2004	25	0	0	14	Female
1978	1999	29	7	2	26	Male
1979	2004	25	0	0	14	Female
1980	2004	34	21	12	22	Female
1981	2003	25	0	9	26	Male
1982	2003	30	14	8	24	Female
1983	2003	29	6	5	24	Male
1984	2002	27	0	0	19	Female
1985	2004	42	14	22	22	Female
1986	2000	44	29	33	30	Female
1987	2005	25	3	1	19	Male
1988	2005	34	13	7	19	Female
1989	2003	50	8	28	27	Male
1990	2000	35	31	17	39	Female
1991	2005	50	13	29	27	Female
1992	2005	33	10	8	25	Female
1993	2005	50	41	57	15	Female
1994	2005	33	3	7	27	Female
1995	2004	38	31	33	25	Female
1996	2004	29	6	4	9	Female
1997	2003	80	57	67	19	Female
1998	2004	33	8	1	29	Female
1999	2005	46	26	22	20	Male
2000	2001	40	49	62	27	Female
2001	2004	27	7	0	11	Female
2002	1998	29	0	0	18	Female
2003	2004	29	37	16	20	Male
2004	2004	25	9	2	19	Female
2005	2002	33	14	0	23	Female
2006	2003	30	22	19	23	Female
2007	2003	61	77	39	17	Female
2008		30	12	11	16	Female
2009	2003	25	0	0	23	Female
2010	2005	32	7	8	21	Female
2011	2004	26	3	0	7	Male
2012	2003	65	46	50	18	Male
2013	2005	76	90	49	18	Female
2014	2005	41	17	14	22	Female
2015	1993	62	7	30	24	Female

2016	2003	42	14	22	24	Male
2017	2004	27	1	16	15	Male
2018	2005	42	20	23	19	Female
2019	2004	47	34	25	16	Female
2020	2005	26	4	17	26	Female
2021	2005	43	39	57	28	Female
2022	2005	25	2	2	16	Female
2023	2004	30	19	9	21	Male
2024	2003	35	16	13	16	Female
2025	2002	39	35	47	17	Female
2026	2002	50	20	28	28	Female
2027	2002	30	10	4	8	Female
2028	2003	33	3	12	13	Male
2029	2004	55	51	48	31	Other (please ty
2030	2004	25	0	7	14	Female
2031	2003	72	43	61	22	Female
2032	2004	54	39	30	23	Male
2033	2003	48	11	38	31	Female
2034	2004	26	6	4	13	Male
2035	1999	42	15	35	27	Female
2036	2003	34	20	9	30	Female
2037	2004	35	7	1	24	Male
2038	2004	28	23	5	28	Male
2039	2005	68	72	28	18	Female
2040	2002	25	3	0	8	Female
2041	2003	36	35	40	16	Male
2042	2004	25	0	7	20	Female
2043	2003	59	47	53	23	Female
2044	2005	85	63	60	21	Female
2045	2003	25	4	2	19	Female
2046	2002	27	19	10	26	Female
2047	2000	34	3	8	15	Female
2048	2004	43	27	16	24	Female
2049	2002	69	35	63	23	Female
2050	2005	50	41	31	21	Female
2051	2001	86	56	36	30	Female
2052	2001	27	0	0	25	Female
2053	2003	26	0	0	16	Male
2054	2000	38	22	47	21	Female
2055	2003	43	31	31	31	Female
2056	2004	40	18	23	19	Female
2057	2005	30	2	2	12	Male

2058	2005	25	12	17	34	Female
2059	2005	36	27	16	26	Male
2060	2004	26	0	0	18	Male
2061		25	5	49	15	Female
2062	2000	28	3	3	23	Male
2063	2005	94	74	60	36	Male
2064	2002	33	21	6	23	Female
2065	2001	69	60	50	34	Female
2066	2004	25	0	12	19	Male
2067	2003	24	3	0	16	Female
2068	2003	25	4	6	16	Male
2069	2003	38	10	33	25	Male
2070	2002	24	0	8	23	Female
2071	2002	29	16	11	15	Female
2072	2005	36	9	19	22	Male
2073	2000	44	30	57	19	Female
2074	2002	39	18	10	21	Female
2075	2004	27	0	0	23	Female
2076	2003	56	16	14	22	Male
2077	2002	29	2	3	21	Female
2078	2005	40	20	35	14	Male
2079	2005	81	82	79	16	Female
2080	2004	53	26	42	22	Female

ype in below):

ype in below):

ype in below):

ype in below):

ype in below):

swer

ype in below):

ype in below):

ype in below):

ype in below):

ype in below):

swer

ype in below):

ype in below):

swer

ype in below):

ype in below):

swer

ype in below):

swer

swer

ype in below):

ype in below):

ype in below):

ype in below):

ype in below):

swer

/pe in below):

ype in below):

ype in below):

ype in below):

ype in below):

ype in below):

ype in below):

ype in below):
