# Supplementary material for: Decreased sound tolerance in a Canadian University Context: Associations with autistic traits, social competence, and gender in an undergraduate sample
Source: PLoS One. 2025 Nov 26;20(11):e0334689. doi: 10.1371/journal.pone.0334689 (PMC12654913; doi:10.1371/journal.pone.0334689)
Supplement: Attachment — Submitted filename: Response To Reviewers.docx [file pone.0334689.s010.docx]

Review Comments to the Author

Reviewer #1: Reviewer comment and suggestions
Generally the findings indicate that misophonia, hyperacusis, and autistic traits are more prevalent among females and non-cisgendered individuals, with significant associations among these variables. While statistically significant, the effect sizes for group differences are often small to moderate, suggesting that individual variability plays a substantial role. Future research could benefit from longitudinal designs, clinical diagnoses, and controlling for additional confounders to deepen understanding.
• The title author need to be revised and study design should be appeared on top of the tittle

**We are uncertain as to what is being requested here.**
• The results you have presented demonstrate thorough statistical analyses and reveal meaningful associations among misophonia, hyperacusis, autistic traits, and social competence across gender groups. They contribute valuable insights to the existing literature. However
• Clarify Limitations: Clearly acknowledge the reliance on self-report measures, the cross-sectional nature, and potential sample biases, especially regarding the small non-cisgender subgroup.

**Thank you for these suggestions. The section discussing the limitations has been expanded to emphasize these points.**
• Strengthen Discussions: Emphasize the practical significance of effect sizes, not just statistical significance, and discuss implications cautiously given the observational design.

**We appreciate this feedback. The discussion has been expanded to include reflection on the effect sizes observed in the study.**

• Ensure Clarity & Detail: Make sure all statistical methods are transparently reported, and figures/tables are well-organized for clarity.

**Figures will be re-uploaded in higher quality to ensure clarity and legibility.**
• Supplementary Materials: Provide detailed supplementary analyses, such as residuals and additional subgroup analyses, to enhance transparency.

**Additional analyses are reported in supplementary materials.**
• If these considerations are addressed, the study appears suitable for publication.

**Thank you, this feedback has helped improve the clarity of our manuscript.**

Reviewer #2: The conclusion is too thin. The author(s) must include other information here not just the summary of the findings. For example, the implication of the study in terms of practice and theory. Also the way it expands our understanding of the concepts and contexts of those that suffer the conditions.

**Thank you for assisting with making the manuscript more robust. More discussion of the findings and their implications has been reiterated from the discussion section to the conclusion section directly.**

Reviewer #3: Dear authors, this is a well-written article. My comments are very minor and technical:
**Nevertheless, thank you. Your feedback is appreciated.**
1. Line 247: including... ? seems like the sentence did not finish.

**This sentence was intended to direct the reader to citation 35, it has been edited to be more clear.**
2. Methodology: Suggest to report the reliability and validity values of each instrument

**These psychometric properties for the relevant measures are currently reported in the supplementary materials. We hope this is satisfactory.**

Journal Requirements:

When submitting your revision, we need you to address these additional requirements.

1. Please ensure that your manuscript meets PLOS ONE's style requirements, including those for file naming. The PLOS ONE style templates can be found at

<https://journals.plos.org/plosone/s/file?id=wjVg/PLOSOne_formatting_sample_main_body.pdf> and [https://journals.plos.org/plosone/s/file?id=ba62/PLOSOne_formatting_sample_title_authors_affiliations.pdf](https://can01.safelinks.protection.outlook.com/?url=https%3A%2F%2Ftrack.editorialmanager.com%2FCL0%2Fhttps%3A%252F%252Fjournals.plos.org%252Fplosone%252Fs%252Ffile%253Fid%3Dba62%252FPLOSOne_formatting_sample_title_authors_affiliations.pdf%2F1%2F010f01992ddfc04b-a4860e95-ad66-4b45-a929-a5d136a73ffb-000000%2FCIvgvlkvOBOZs0dzOEA3Ur05exLiSPoFFpWWlFu93vI%3D227&data=05%7C02%7Cmann7000%40mylaurier.ca%7C4a25473aa3674b2a0b1a08ddf0451d1a%7Cb45a5125b29846bc8b89ea5a7343fde8%7C1%7C0%7C638930901830333259%7CUnknown%7CTWFpbGZsb3d8eyJFbXB0eU1hcGkiOnRydWUsIlYiOiIwLjAuMDAwMCIsIlAiOiJXaW4zMiIsIkFOIjoiTWFpbCIsIldUIjoyfQ%3D%3D%7C0%7C%7C%7C&sdata=k%2BOiGMvt5EqDl4JboZNM65m8rd%2BPum3IW3PG2qwiAbw%3D&reserved=0)

**We have done our best to modify the document to align with the linked style guide, though the guide does not provide information about how to format level 4 headings.**

2. Please update your submission to use the PLOS LaTeX template. The template and more information on our requirements for LaTeX submissions can be found at <http://journals.plos.org/plosone/s/latex>.

**We are not familiar with LaTeX. We have reached out to PLOS ONE, but we did not receive a response. For the moment, we have resubmitted using a PDF file. Please advise.**

3. We note that the grant information you provided in the ‘Funding Information’ and ‘Financial Disclosure’ sections do not match.

When you resubmit, please ensure that you provide the correct grant numbers for the awards you received for your study in the ‘Funding Information’ section.

**This research was supported by the Wilfrid Laurier University Research Support fund that helps to support all faculty and students at Laurier (i.e., SM, NV, NS). There is no specific grant number. The funders had no role in study design, data collection and analysis, decision to publish, or preparation of the manuscript.**

4. Thank you for stating in your Funding Statement:

[Wilfrid Laurier University	[http://dx.doi.org/10.13039/100012531](https://can01.safelinks.protection.outlook.com/?url=http%3A%2F%2Ftrack.editorialmanager.com%2FCL0%2Fhttp%3A%252F%252Fdx.doi.org%252F10.13039%252F100012531%2F1%2F010f01992ddfc04b-a4860e95-ad66-4b45-a929-a5d136a73ffb-000000%2FMoO_S2Idd7N0ydErxUhkRTfuNYm9nK-m6kMV2W03Jnw%3D227&data=05%7C02%7Cmann7000%40mylaurier.ca%7C4a25473aa3674b2a0b1a08ddf0451d1a%7Cb45a5125b29846bc8b89ea5a7343fde8%7C1%7C0%7C638930901830368412%7CUnknown%7CTWFpbGZsb3d8eyJFbXB0eU1hcGkiOnRydWUsIlYiOiIwLjAuMDAwMCIsIlAiOiJXaW4zMiIsIkFOIjoiTWFpbCIsIldUIjoyfQ%3D%3D%7C0%7C%7C%7C&sdata=c7ze8mEX5Y0gCU4bGHRckFwsU4DiDZcwIrV%2Bto64odo%3D&reserved=0)	Research Support Fund	Dr Nichole Scheerer].

Please provide an amended statement that declares *all* the funding or sources of support (whether external or internal to your organization) received during this study, as detailed online in our guide for authors at <http://journals.plos.org/plosone/s/submit-now>. Please also include the statement “There was no additional external funding received for this study.” in your updated Funding Statement.

Please include your amended Funding Statement within your cover letter. We will change the online submission form on your behalf.

**See above. This has been added to the cover letter.**

5. In the online submission form you indicate that your data is not available for proprietary reasons and have provided a contact point for accessing this data. Please note that your current contact point is a co-author on this manuscript. According to our Data Policy, the contact point must not be an author on the manuscript and must be an institutional contact, ideally not an individual. Please revise your data statement to a non-author institutional point of contact, such as a data access or ethics committee, and send this to us via return email. Please also include contact information for the third party organization, and please include the full citation of where the data can be found.

**After speaking with our REB, we have been granted permission to include the deidentified data as supplementary. It has now been attached to the submission.**

6. We note that you have referenced ([35] Wickie A, Van Esch N, Scheerer NE, Social success in a noisy world: Exploring the relationship between decreased sound tolerance and social profiles. Unpublished.) which has currently not yet been accepted for publication. Please remove this from your References and amend this to state in the body of your manuscript: (ie “Bewick et al. [Unpublished]”) as detailed online in our guide for authors

<http://journals.plos.org/plosone/s/submission-guidelines#loc-reference-style>

**This manuscript has been published. The citation has been modified to reflect this.**

7. Please upload a new copy of Figures 1, 2, 3, and 4 as the detail is not clear. Please follow the link for more information: <https://blogs.plos.org/plos/2019/06/looking-good-tips-for-creating-your-plos-figures-graphics/>

**We have reuploaded the figures in higher resolution.**

8. Please include a separate caption for each table in your manuscript.

**These captions have been added.**

9. Please include captions for your Supporting Information files at the end of your manuscript, and update any in-text citations to match accordingly. Please see our Supporting Information guidelines for more information: <http://journals.plos.org/plosone/s/supporting-information>.

**Captions have been added to the end of the manuscript.**
